# Reproductive Efficiency in Sheep: Estimates and Relationships with Fertility, Fecundity and Lamb Survival

**DOI:** 10.3390/ani16111608

**Published:** 2026-05-25

**Authors:** David O. Kleemann, Alyce M. Lowe, Alice C. Weaver

**Affiliations:** Aquatic and Livestock Sciences, South Australian Research and Development Institute, Turretfield Research Centre, Rosedale, SA 5350, Australia; alyce.lowe@sa.gov.au (A.M.L.); alice.weaver@sa.gov.au (A.C.W.)

**Keywords:** sheep, reproductive efficiency, fertility, fecundity, lamb survival

## Abstract

Addressing increased demand for sheep meat globally is dependent on reproductive efficiency (lambs weaned per 100 breeding ewes). Efficiency is determined by ewe fertility (pregnancy rate) and fecundity (litter size), as well as lamb survival. This paper provides estimates of these components from the world literature and examines their relationships with breed class (wool, dual-purpose, meat, milk, prolific) and within breed class, country and global region. Reproductive efficiency was estimated at 97.7 ± 2.68, while values for fertility, fecundity and lamb survival were 83.9 ± 0.95, 144.6 ± 3.48 and 80.8 ± 0.93%, respectively. Efficiency increased for each component, with the response being greatest for fecundity (R^2^ = 0.4896, *p* < 0.001). Within breed classes, medium- and strong-wool Merinos were more (*p* < 0.05) efficient than fine-wools, while narrow-tailed meat breeds were more fecund (*p* < 0.05) than fat-tail, wool-shedders and hair types. Efficiencies in the single- versus multiple-gene prolific groups were 124.6 ± 32.52% and 152.9 ± 10.1%, respectively. In conclusion, these findings provide an overview for the reproductive efficiency of the world’s sheep population (though based on <5% of breeds), quantify how the parameters controlling efficiency interact, and estimate contributions made by breed, country and world region toward reproductive output.

## Simple Summary

Enhanced reproductive efficiency is key to meeting the challenge of the increased demand for sheep meat globally. This can be achieved by improving the pregnancy rate (fertility) and litter size (fecundity) of ewes and the survival of their lambs. Predicting how these parameters interact is important to achieve optimal reproductive efficiency (number of lambs weaned per breeding ewe). The aim was to establish mean values for these parameters and model relationships from world data. Gains in reproductive efficiency increased for each parameter (fertility, fecundity, and survival), but major gains that were made by improving fecundity were limited by reduced survival. Specialist ‘wool’-producing sheep were the least efficient, compared with the ‘prolific’ class, which were the most efficient. Efficiency values for other classes (dual-purpose, meat, milk, fat-tail, wool-shedders) were intermediate. Benchmarking of major breeds within countries for reproductive efficiency and its parameters focused research and educational effort on the parameters that needed the most improvement and was successful. In conclusion, this study provides an overview of the reproductive efficiency of the world’s sheep population (though based on <5% of global breeds), establishes how the parameters controlling efficiency interact, and estimates contributions made by breed, country and world region toward reproductive output.

## 1. Introduction

The domestication of sheep by man has a long 10,000-year history, with the species now widespread globally [1]. The world’s sheep population numbers about 1.3 billion, with dominance in Asia, Africa and Oceania and lesser numbers in Europe and the Americas [2]. They play an important role in providing human nutrition (meat, milk and cheese) and fibre for clothing and other uses. Numbers have increased substantially over the past two decades in developing countries and sheep continue to play a significant role in the developed world [2]. Although demand for sheep products is expected to increase, particularly meat [3], their long-term prospects will depend on competition from other meat sources [3], climate change and animal health factors [4].

Meeting the increased demand for sheep meat production is dependent on socio-economic, cultural and biological factors, with the most important factors affecting the latter likely to be reproduction rates and nutrition. While the supply of feed ultimately determines the number of breeding ewes which can be grazed or housed, it is the reproductive rate that has a large bearing on the efficiency of production [5,6]. For example, the biological efficiency of meat production that was adjusted for feed intake increased by 50–55% when ewes raised two compared with one lamb to weaning [7]. Given that reproductive rate is the key driver of biological efficiency, for the purposes of this paper, efficiency will be defined as the number of lambs present at weaning per breeding animal. Reproductive efficiency defined in this manner consists of three components: fertility (pregnancy rate), fecundity (lambs per pregnancy or litter size) and lamb survival. The product of these components gives the number of lambs weaned per ewe exposed to rams [8].

While reproductive efficiency values for different sheep breeds were identified to some extent in earlier reports [9,10,11], a comprehensive listing of scientific papers that include additional breeds as well as those bred for high fecundity [12] is required to establish values for reproductive efficiency and its components across all breeds and allow the examination of the relationships between them. The relationship between fertility and fecundity has been established from within-flock data [13], indicating that pregnancy rate improves with increased prolificacy; estimates from between-flock data were not reported. Similarly, the negative relationship between lamb survival and fecundity is well documented within breeds [14,15], yet the extent of response across breeds and within breed classes (wool, dual-purpose, meat) is not established on a global scale. Furthermore, information on reproductive efficiency and its three components for sheep is required on a broad scale to evaluate and predict different scenarios for increasing the world supply of sheep meat to meet perceived demand.

This review determines estimates of reproductive components (fertility, fecundity, lamb survival) and resultant reproductive efficiency and provides relationships between the components from data collated from the world’s scientific literature. In addition, the effect of breed class (wool, wool-and-meat or dual-purpose, meat, milk, prolific), specific breeds, country and world region on the reproductive components was assessed to give a global perspective on the reproductive efficiency of sheep.

## 2. Materials and Methods

### 2.1. Protocol and Registration

A protocol describing the objectives, eligibility criteria, search strategy, study selection procedures, data extraction items and planned analyses was developed but was not formally registered in a public repository (Table S1). The review was conducted and reported in accordance with the PRISMA 2020 and PRISMA-S guidelines to ensure transparency and reproducibility [16,17].

### 2.2. Eligible Criteria

Studies were eligible for inclusion when they reported all three key reproductive components—fertility (ewes pregnant per 100 ewes exposed to rams), fecundity (lambs born per 100 ewes pregnant) and lamb survival (lambs weaned per 100 lambs born)—allowing the calculation of reproductive efficiency. In addition, studies were considered when means were derived from mature age ewes and from ewes 1–2 years old at first mating, when available, and where experimental or observational designs were employed in which flock- or group-level mean values were reported. Finally, emphasis was placed on the inclusion of datasets that contained large numbers of animals, as commonly found in studies examining genetic parameters, and where ewes were mainly subjected to an annual mating system (Table S2). Studies that involved artificial insemination, estrus synchronisation protocols or other artificial reproductive technologies were not included, as most did not meet the eligibility criteria (Table S1).

### 2.3. Breed and Region Classifications

Breeds were classified following Fogarty (1995) [9], Safari and Fogarty (2003) [10], and Safari et al. (2005) [11], with additional subdivisions for wool (fine, medium, strong, unspecified), meat (short- and long-narrow-tail; fat-tail; wool-shedders; hair), milk (short-/long-tail; fat-tail), and prolific (multiple-gene vs. FecB single-gene) categories. A dual-purpose class, as defined by Fogarty (1995) [9], was included for breeds bred for both meat and wool. Countries were grouped into seven world regions following Our World in Data (2018) [18]: North America, South America, Europe/Central Asia, Middle East/North Africa, Sub-Saharan Africa, South Asia and East Asia/the Pacific.

### 2.4. Special Inclusion Rules and Exclusion Criteria

Studies with small sample sizes (*n* < 50 ewes) were included where such records provided otherwise unavailable breed or genotype information. When a reproductive component was missing, it was calculated from other components where possible (for example, lamb survival = lambs weaned/(fertility × fecundity).

We excluded studies lacking at least three reproductive components, studies on species other than sheep, review/concept papers, conference abstracts without extractable numerical data and non-English language publications due to resource constraints (noting the potential for language bias).

### 2.5. Information Sources

The initial dataset was derived from review papers that contained comprehensive reference lists of genetic studies that included the parameters for the three reproductive components (fertility, fecundity, lamb survival) as well as reproductive efficiency (lambs weaned of ewes exposed to rams) in sheep up to and including 2003 [9,10,11]. An additional literature search was conducted in Web of Science to extend coverage, focusing on studies published from 2004 up to and including 2024.

Several studies not retrieved through database searches (*n* = 9) but known to the authors were included to ensure representation of key breeds and reproductive genotypes (particularly high-fecundity lines).

### 2.6. Search Category

The search strategy was limited to terms that were likely to define large data sets (genetic parameters) and to select those with reproductive data (reproduction) in the ovine species (sheep). A Boolean operator (“AND”) was applied.

Search terms included sheep, genetic parameters and reproduction, and was limited by date (1 January 2004 to 30 November 2024) and by Title/Abstract (see Table S3).

### 2.7. Study Section

**Figure 1:**
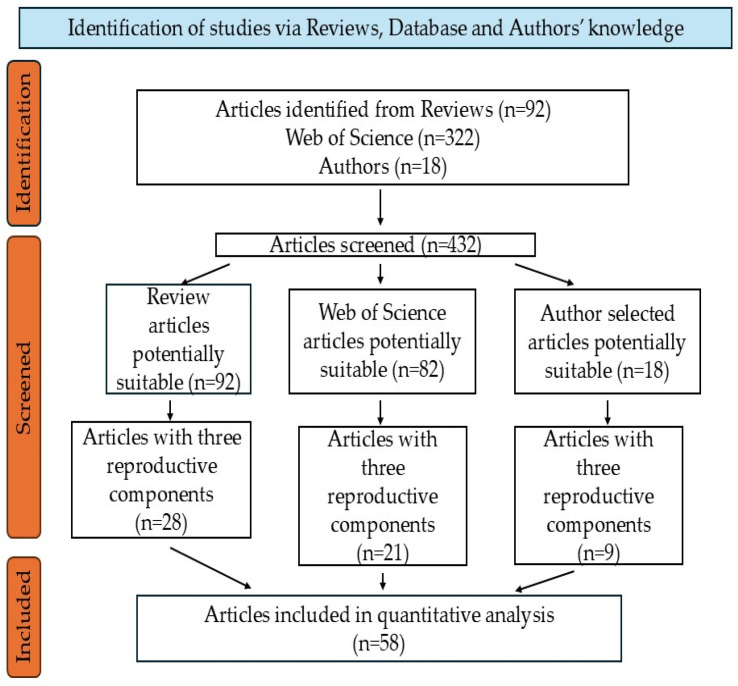
PRISMA Flow Chart.

A total of 410 reports were identified across all information sources. After screening titles, abstracts and full texts, 112 studies met the inclusion criteria by reporting all three reproductive components (Figure 1).

Screening was conducted by one reviewer (D.O.K.). Reasons for exclusion of full-text articles are detailed in Table S2.

The study selection process is summarised in a PRISMA 2020 flow diagram (Figure 1) [16,17].

### 2.8. Data Extraction

All extracted values were subsequently checked for internal consistency against the original publications. For each included study, the following information was extracted:

1. fertility, fecundity, lamb survival, and lambs weaned per 100 ewes exposed to rams.
2. sample size.
3. breed and breed category (including prolificacy gene classification).
4. country and world region.
5. mating system.
6. ewe age.

Where a reproductive component or reproductive efficiency was not reported but could be derived from other components and/or reproductive efficiency, calculated values were used.

### 2.9. Risk of Bias Assessment

Risk of bias was assessed using the SYRCLE tool (Radboud University Medical Centre, Nijmegen, The Netherlands), adapted for livestock studies. Domains included selection bias, performance bias, detection bias, attrition bias, reporting bias and other potential biases (e.g., nutritional or environmental variation).

Each domain was classified as low, unclear or high risk, with full assessments provided in Table S5.

### 2.10. Data Synthesis and Statistical Analysis

A structured quantitative synthesis was performed using IBM SPSS Statistics Version 26 (IBM Corp., Armonk, NY, USA). Descriptive statistics were calculated for each reproductive component and for reproductive efficiency. Associations between components and with reproductive efficiency were examined using linear, exponential and polynomial regression models, with model choice based on R^2^ improvements using curvilinear procedures. Differences across breed classes, breed groups within classes, countries and world regions were assessed using General Linear Models (Univariate) with Weighted Least Squares, and Bonferroni-adjusted post hoc comparisons. Significance was set at *p* ≤ 0.05.

To assess sensitivity to study weighting, all general linear models were re-run without weights and compared with a model weighted by number of animals per record. Estimated marginal means were extracted for each breed class under both approaches.

In addition to the study-level analysis reported above, we conducted formal random-effects meta-analysis and meta-regressions to quantitively synthesize reproductive outcomes across studies to explore sources of heterogeneity.

Fertility and survival were analysed as proportional outcomes using logit-transformed random-effects models, whereas fecundity and number of lambs weaned were analysed as continuous outcomes because values frequently exceeded 100%. Random-effects models were fitted using restricted maximum likelihood (REML) estimations. Between-study heterogeneity was quantified using τ^2^ and I^2^ statistics.

Meta-regression models were used to assess the influence of year of publication, world region and breed class on reproductive outcomes. Wool breeds and Europe were used as reference categories for breed class and geographic comparisons, respectively. The proportion of heterogeneity explained by moderators was summarized using R2 statistics and Wald-type QM tests. All analyses were conducted in R using the *metafor* package.

## 3. Results

### 3.1. Overall Descriptive Statistics

Results for each reproductive component across all sheep breeds (*n* = 51) are summarised in Table 1. These findings were based on 58 articles contributing 112 records where all components were measured. On average, 84% of ewes mated became pregnant, with a range from 42% to 98%. Each pregnancy produced 1.45 lambs at birth, with values ranging from 1.02 to 2.68 lambs, reflecting substantial variation in fecundity (litter size) within the global sheep population. Lamb survival to weaning averaged 81%, indicating that approximately 20% of lambs born succumb to the birth process and other vagaries prior to achieving independence from their mother’s support to weaning. The combined effect of these components provides an estimate of reproductive efficiency, with an average of 97.7 lambs weaned per 100 ewes exposed to rams. However, efficiency varied widely among studies, ranging from 19 to 192 lambs present at weaning.

### 3.2. Relationships Between Reproductive Components and Reproductive Efficiency

**Figure 2:**
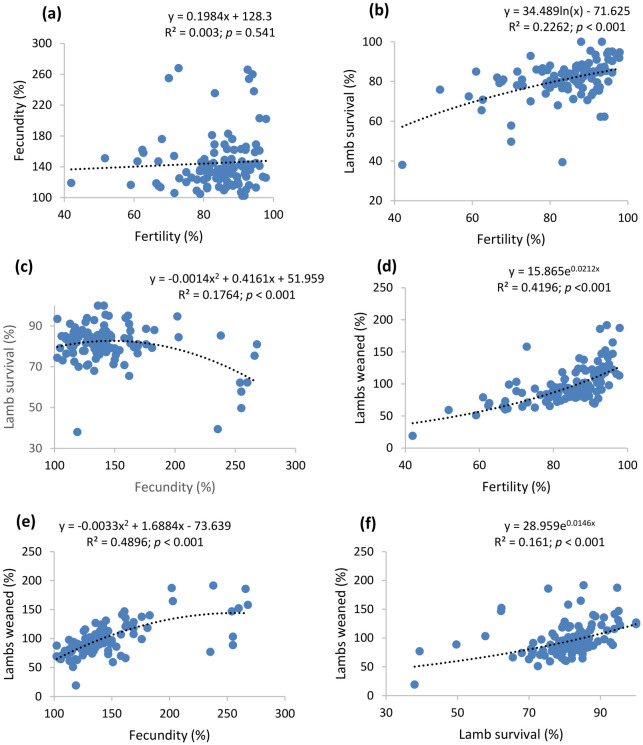
(**a**–**f**) Relationships between reproductive components (fertility—ewes pregnant of ewes exposed to rams: fecundity—lambs born of ewes pregnant; lamb survival—lambs present at weaning of lambs born) and with reproductive efficiency (lambs weaned of ewes exposed to rams).

Relationships between the reproductive components are illustrated in Figure 2a–f. There was virtually no relationship between fecundity and fertility (Figure 2a; R^2^ = 0.003; *p* = 0.541) while lamb survival increased logarithmically as fertility increased (Figure 2b; R^2^ = 0.226; *p* < 0.001). Lamb survival decreased quadratically as fecundity increased (Figure 2c; R^2^ = 0.176; *p* < 0.001). Reproductive efficiency (lambs weaned) increased exponentially (*p* < 0.001) when fertility and lamb survival increased (Figure 2d,f; R^2^ = 0.420 and 0.161, respectively). In addition, reproductive efficiency increased quadratically as fecundity increased (Figure 2e; R^2^ = 0.490; *p* < 0.001).

### 3.3. Breed Class Means and Relationships of Reproductive Components

Breeds were categorised according to their primary purpose: wool, meat, milk, dual-purpose (similar emphasis on wool and meat), and prolific (greater than approximately two lambs per pregnancy). Results indicated that dual-purpose sheep exhibited greater fertility (5–6%; *p* < 0.05) and fecundity (13–18%; *p* < 0.05) compared with wool and meat classes (Table 2; Supplementary Figure S1). Fertility did not differ among milk, prolific, wool, meat or dual-purpose classes; however, the fecundity of prolific sheep was 99–116% higher (*p* < 0.05) than all other classes. In contrast, lamb survival was higher for meat sheep compared with the wool and dual-purpose classes (7–9%: *p* < 0.05) and did not vary with milk and prolific groups. Overall, reproductive efficiency (lambs weaned of ewes exposed to rams) was 54–68% greater (*p* < 0.05) in prolific breeds compared with other classes, whereas wool breeds exhibited the lowest efficiency at 81.5%. The difference in efficiency between the wool and prolific groups was primarily due to the large variation in fecundity, with relatively minor variations in fertility and survival.

**Figure 3:**
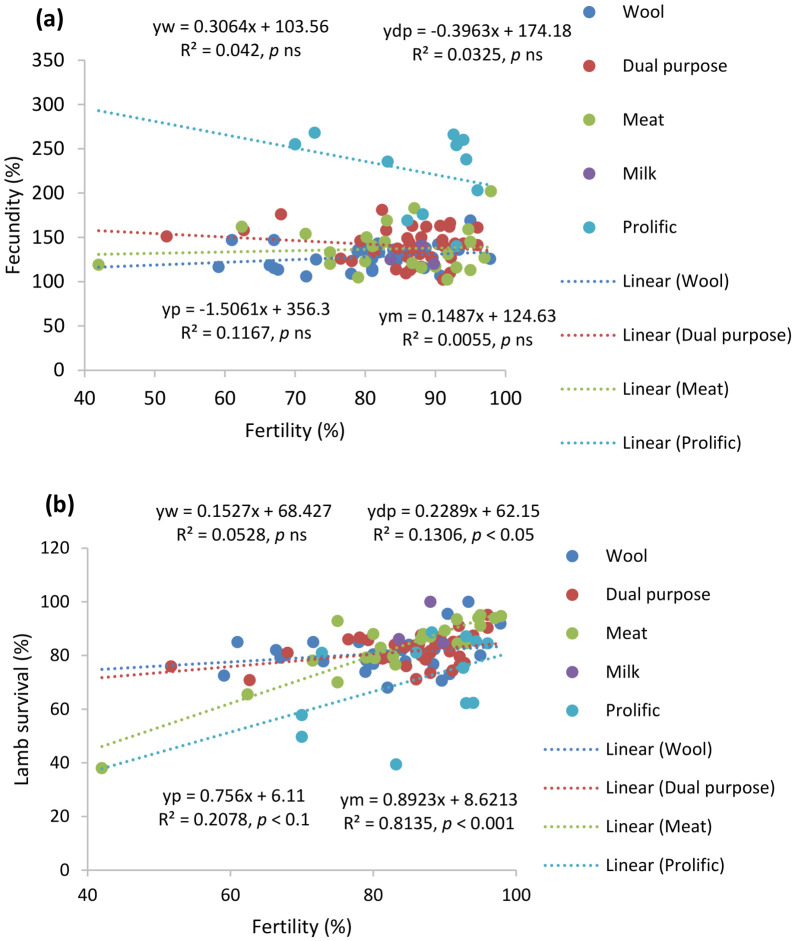

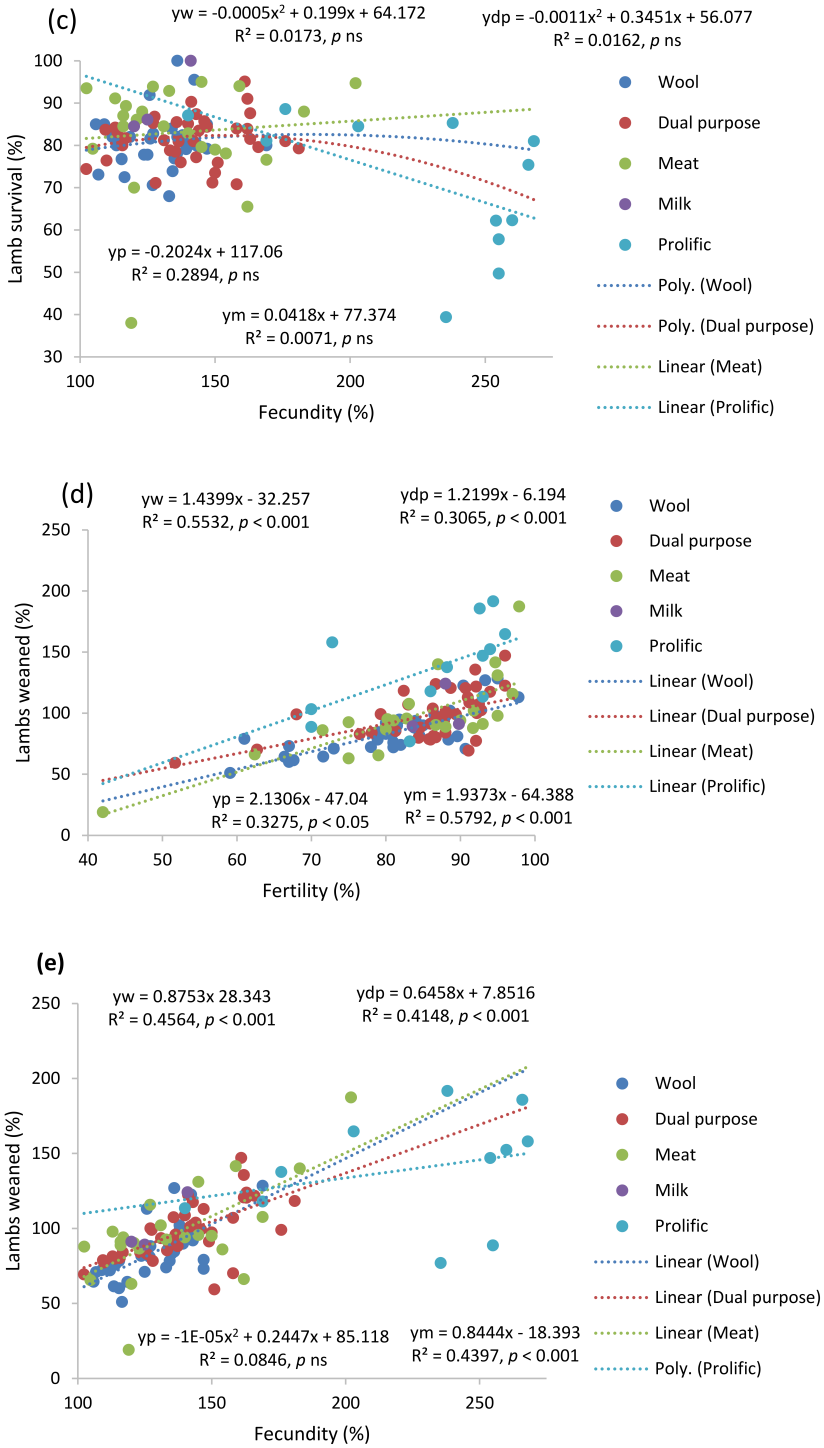

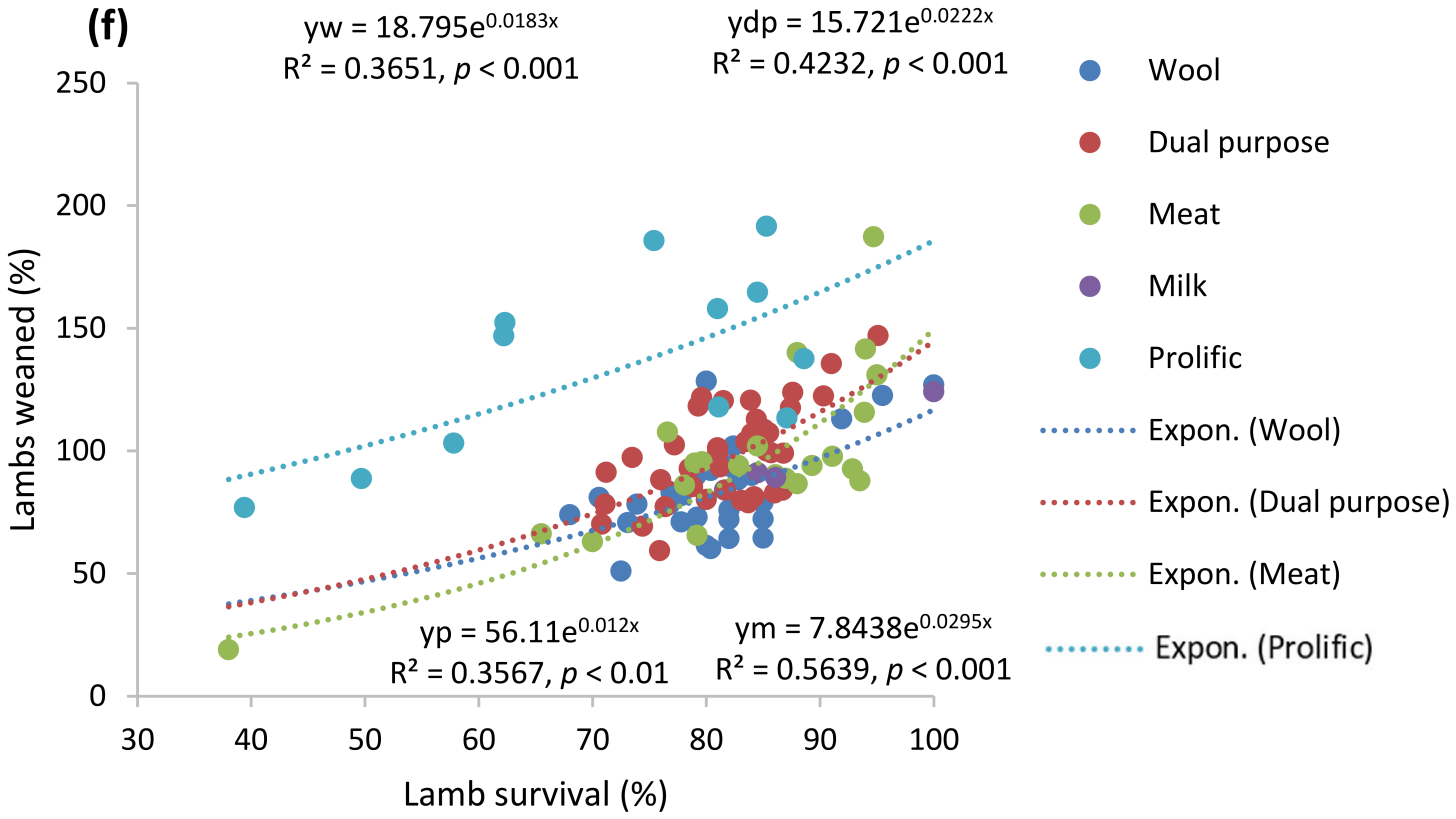
(**a**,**b**) Relationships between reproductive components (fertility—ewes pregnant of ewes exposed to rams: fecundity—lambs born of ewes pregnant; lamb survival—lambs present at weaning of lambs born) and with reproductive efficiency (lambs weaned of ewes exposed to rams) within breed classes. (**c**,**d**) Relationships between reproductive components (fertility—ewes pregnant of ewes exposed to rams: fecundity—lambs born of ewes pregnant; lamb survival—lambs present at weaning of lambs born) and with reproductive efficiency (lambs weaned of ewes exposed to rams) within breed classes. (**e**,**f**) Relationships between reproductive components (fertility—ewes pregnant of ewes exposed to rams: fecundity—lambs born of ewes pregnant; lamb survival—lambs present at weaning of lambs born) and with reproductive efficiency (lambs weaned of ewes exposed to rams) within breed classes.

Relationships between reproductive components were examined within breed classes for four of the five classes (wool, dual-purpose, meat, prolific) with milk enterprise not examined due to the small number of reports (*n* = 3). Data points are presented graphically for all breed classes including milk, while equations and trendlines are presented for the other breed classes only (Figure 3a–f).

No significant relationships were observed between fecundity and fertility within breed classes (Figure 3a). Lamb survival increased with increasing fertility for the dual-purpose (*p* < 0.05) and meat (*p* < 0.001) classes, while the prolific class showed a weak yet non-significant (*p* < 0.1) association (Figure 3b). No significant relationships were detected between lamb survival and fecundity within breed classes (Figure 3c). In contrast, reproductive efficiency (lambs weaned) was positively associated with all reproductive components across all breed classes (Figure 3d–f). Most relationships were best described as linear, although some were better fitted as polynomial (Figure 3c,e) or exponential (Figure 3f).

To assess sensitivity of study size on breed-class comparisons, all general linear models were re-run without weighting and compared with models weighted by the number of animals represented per record. Weighting altered the magnitude of some estimates but did not change the relative ranking or interpretations of breed class for any reproductive component. Prolific breeds remained clearly distinct from other classes for fecundity and reproductive efficiency, while differences among wool, meat and dual-purpose classes were consistent across weighting approaches. Full weighted and unweighted model outputs, including estimated marginal means, are provided in Table S6a,b.

### 3.4. Groups Within Breed Classes

Within the wool class, fine-wool breeds exhibited lower reproductive efficiency compared with the medium- and strong-wool types (14–16%), although differences were not statistically significant (Table 3; Supplementary Figure S2). The observed numerical difference was primarily attributed to fecundity, which was greater (*p* < 0.05) for the coarser-wool types.

Among meat types, those with short and long narrow-tails were more fecund (*p* < 0.05;) and showed greater reproductive efficiency (15–25%) compared with fat-tail, wool-shedders or hair types.

The milk class was represented by three experimental reports that included data for all reproductive components. This class comprised of two fat-tail and one narrow-tailed breed (Table 3). Results indicated low efficiency (76–86%), primarily due to low fecundity (108–125%) and lamb survival (76–86%).

Prolific breeds in which fecundity is influenced by multiple genes appeared more efficient than those where fecundity is controlled by a single gene (153% vs. 125%), although this difference was not statistically significant (Table 3). The observed numerical difference was largely due to higher lamb survival in breeds where prolificacy is controlled by many genes.

### 3.5. Breed

Results for reproductive components by breed are presented in order of declining reproductive efficiency (Table 4) and alphabetically (Supplementary Table S1; Supplementary Figure S3). References for the breeds given below can be found in Table 4. The most efficient breeds were generally those exhibiting high fecundity, including D’man, Rambouillet, Perendale and Romney carrying the *Fec* gene, Flevolander introgressed with Finn breed genetics and Romanov. For these breeds, lambs weaned per 100 ewes exposed to rams ranged from 138 to 192%. In contrast, the least efficient breeds (<90%), were typically associated with low fecundity, such as Merino, Rambouillet, Polwarth, Dorper and Scottish Blackface. An exception was the Merino heterozygous for the *FecB* gene, which displayed high fecundity (236%), yet poor lamb survival (39%) resulting in low reproductive efficiency (77%). Breeds with intermediate efficiency (90–130%) included Romney, Corriedale, Targhee, Dorset and Turcan. The Finn breed fell within this intermediate range (99%) which was surprising given its high fecundity (253%), but this reflects its poor performance in both fertility (71%) and lamb survival (55%).

### 3.6. Country

Results for reproductive components and reproductive efficiency by country are given in Table 5 and Supplementary Figure S4. References for countries mentioned below can be found in Table 5. Comparisons across countries indicated the disproportionate number of experiments reported from Australia, New Zealand and the United States of America (U.S.) compared with other countries. The efficiency of sheep flocks in New Zealand was greater (112%; *p* < 0.05) than those in the U.S. (89.2%) and Australia (83.9%). Interestingly, fertility and lamb survival were greater (*p* < 0.05) and fecundity tended to be higher in New Zealand compared with Australia. Though fecundity in U.S. flocks was reasonably high (143%), a comparatively low lamb survival (73%) was the main contributor to reduced efficiency compared with New Zealand. In contrast, lower fecundity and higher lamb survival (*p* < 0.05) of flocks in South Africa compared to those in the U.S. were the main contributors to a similar efficiency for these countries (87% versus 89%, respectively). Another country with a reasonable number of reports (*n* = 9) was Turkey, with flocks of high fertility and high survival (>90%) but low fecundity (124%) contributing to a moderate efficiency (106%).

### 3.7. World Regions

On a world-region basis, fecundity was higher (*p* < 0.05) in North America (143%) compared with Sub-Saharan Africa (123%), while the reverse was evident for lamb survival (73% versus 88%, respectively; *p* < 0.05), with little difference between these regions for reproductive efficiency (89% vs. 87%, respectively (Table 6; Supplementary Figure S5). Flocks in Europe were the most efficient numerically (110%) compared with the South American region, which had the lowest efficiency (68%; *p* > 0.05), while flocks in the Middle East/North Africa were intermediate (95%). Flocks in East Asia (principally Australia and New Zealand) and North America were also intermediate for efficiency (88% and 89%, respectively).

### 3.8. Meta-Regression Analysis

The meta-regression analysis was highly consistent with the main results presented above. Regarding fertility, a multivariate random-effects meta-regression including year of publication, world region and breed class was statistically significant and explained 20.9% of the between-study heterogeneity, reducing τ^2^ from 0.47 to 0.36. Year of publication was not associated with fertility outcomes. In contrast, fertility was lower in North America (*p* = 0.0006), East Asia/the Pacific (*p* = 0.049) and South America (*p* = 0.013) compared to Europe. Breed class alone explained little heterogeneity and was not retained in the final fertility model, although dual-purpose breeds exhibited slightly higher fertility compared to wool breeds when analysed jointly within year and region.

Meta-analysis of fecundity revealed extreme heterogeneity, with meta-regression explaining 74.9% of between-study variation. Fecundity increased significantly over time. Compared with wool breeds, the dual-purpose and meat breed classes showed moderately higher fecundity (~13 to 14 additional lambs per 100 ewes), while the highly prolific breed class exhibited substantially greater fecundity (>100 additional lambs per 100 ewes). World region also influenced fecundity, with higher values reported in North America and East Asia/the Pacific. Random-effects meta-analysis indicated a mean survival of approximately 81% with substantial heterogeneity. Meta-regression explained 41.0% of between-study variation. Survival did not show a consistent temporal trend after adjustment. Survival was significantly lower in North America, East Asia/the Pacific and South America compared to Europe. Highly prolific breeds also exhibited reduced survival relative to wool breeds, consistent with trade-offs between productive output and longevity.

Meta-analysis of number of lambs weaned (NLW) revealed substantial heterogeneity, with an average of ~98 lambs weaned per 100 ewes. Meta-regression explained 41% of heterogeneity. NLW increased significantly over time. Compared with wool breeds, dual-purpose breeds weaned around 21 additional lambs per 100 ewes, while highly prolific breeds weaned approximately 50 additional lambs per 100 ewes. NLW was lower in South America and East Asia/the Pacific relative to Europe.

## 4. Discussion

### 4.1. Global Perspective on Sheep Reproductive Efficiency

This study evaluated the reproductive efficiency of sheep by examining data records collected over the past eight decades as reported in scientific journals from countries all over the world. The present study estimated reproductive efficiency across all breeds to be 99.6 lambs weaned per 100 ewes exposed to rams. While it was anticipated that a preponderance of records would come from countries where research activity was established for a long time, there are a few reports from more recent decades that have broadened the information available regarding country of origin and breed substantially. In addition, from a global perspective, the current evaluation of reproductive efficiency is limited when considering that <5% of the world’s breeds are represented in the present evaluation (51 versus 1155 breeds; [77]. This is the first time that relationships of reproductive efficiency with its three components (fertility, fecundity, lamb survival) have been established from a set of data produced from global information. All three components contributed positively to efficiency (Figure 2e–f), indicating the importance of attending to all components when studying the overall reproductive system. While steady gains in efficiency could be expected from improving both fertility and lamb survival (Figure 2d,f, respectively), incremental gains in efficiency declined as fecundity increased, particularly after fecundity had reached 200% (Figure 2e). This relative decline in efficiency reflects an ever-increasing reduction in lamb survival as fecundity increases (Figure 2c). When fecundity increased from 200% to the point of inflexion at 255%, efficiency increased marginally from 132% to 142%, respectively. In other words, for an additional 55 lambs that were born, only 10 were weaned. Obviously, lamb survival becomes a major issue, from both a production and welfare perspective, as fecundity reaches more than 200%. At this level of fecundity and beyond, the proportion of triplet and higher-order births increases substantially, resulting in significantly lower survival rates compared with births of twins and singles. While Kenyon et al. (2019) [15] have thoroughly reviewed the factors controlling triplet survival and proposed several areas of research for the benefit of extensively grazed flocks, there was one impediment preventing progress that was worth considering. There is wide variation in the birth weight of triplets, which probably results from the competition of the embryos for implantation sites that ensues during migration in the uterus prior to placentation, as well as the mortality of some embryos resulting in variation in nutrient supply during gestation [78,79] This variation causes many downstream consequences that are unfavourable for survival of the triplets (and higher-order births), as shown by Kenyon et al. (2019) [15]. Certainly, improving the survival of offspring as fecundity increases above levels of 200% has a major impact on reproductive efficiency.

The relationships between the components themselves, such as the association between fertility and fecundity, between fertility and survival and between fecundity and survival (as given in Figure 2a–c, respectively), were similar in direction to those calculated from the study of Kleemann and Walker (2005a, b) [65,80]. In their large-scale study, comprised of an evaluation of reproductive parameters in 68 commercial Merino flocks, corresponding relationships between the above components were as follows: y = 0.4181x + 89.466 (R^2^ = 0.0427, *p* < 0.10), y = 39.935ln(x) − 106.8 (R^2^ = 0.1699, *p* < 0.001) and y = −15.25 ln(x) + 146.25 (R^2^ = 0.0388, *p* = 0.10). These equations and those of the current study may become useful when predicting the impacts of global environmental shifts on reproductive efficiency and its components. For example, equations calculating the effect of increased ambient temperature on ewe fertility [80,81] were used to predict the effects of various climate change conditions on reproduction of the Australian sheep flock [82,83].

While these relationships for reproductive efficiency are based on data that includes all sheep breeds, the question remains whether the relationships change when the data is categorised according to breed class (wool, dual-purpose, meat, milk, prolificacy).

The meta-regression analysis (Section 3.8 and Table 7) provided formal statistical support for the descriptive pattern identified in the review. Across reproductive components, breed class and world region explained a substantial proportion of between-study heterogeneity, confirming that variation in reproductive efficiency is not random but is systematically associated with genetic type and production context. The highest proportion of explained heterogeneity was observed for fecundity, indicating that the difference among breed classes and regions accounts for much of the global variation in litter size, whereas fertility showed comparatively limited heterogeneity and weaker moderator effects. Lamb survival displayed intermediate behaviour, with region and breed class explaining meaningful but incomplete variation, consistent with its strong dependence on management and environment.

Importantly, the meta-regression identify contradiction to the global relationships describes above. Instead, it reinforced the conclusion that increased fecundity drives gains in reproductive efficiency only up to a point, beyond which declining lamb survival increasingly constrains efficiency. The persistence if these patterns after accounting for breed class, region and time (as measured by year of publication), indicates that the biological trade-offs between fecundity and lamb survival are fundamental characteristics of the sheep reproductive system, rather than artefacts of study design or regional bias.

### 4.2. Breed Classes

Relationships of reproductive efficiency with their components were similar between all breed classes except prolificacy (Figure 3e,f). In the latter case reproductive efficiency was higher at all levels of fertility and lamb survival compared with relationships of other breed classes. Reasons for these relationship differences for fertility was possibly due to higher numbers of embryos from increased ovulation rates of the prolific breeds improving fertility rate *per se*. This effect was demonstrated for the low fecund Merino [80,81] and highly fecund Romanov [84] yet was not apparent for the prolific class in the current study (Figure 3a). Secondly, relationship differences between prolific and other breed classes for lamb survival may be of genetic origin where prolific breeds display, for example, better mothering ability [85] and where improvement in survival may be due to better environmental management [86].

Interestingly, lamb survival increased with increasing fertility in the prolific and meat groups, responding more in these than in the wool and dual-purpose groups (Figure 3b). This relationship can be explained by a positive relationship between both lamb survival (singles and twins) and fertility and live weight and body condition at mating for the Merino type [80] It is speculated that the lower lamb survival of the prolific group at low fertility compared with the wool and dual-purpose groups may be due to differences between the groups in body condition at mating. An explanation for the response of the positive relationship between survival and fertility of the meat group being different to that of the wool and dual-purpose groups could be due to a possible outlier at the lower end of the relationship having an undue influence on the slope of the line (Figure 3b).

The mean values for reproductive efficiency of the breed classes varied, with wool being the least efficient (82%) followed by meat, milk and dual-purpose (87%, 92% and 96%, respectively), and the prolific class being the most efficient (150%). For example, efficiencies of the Merino wool breed were low at 83% [68] and 84% [62], while efficiencies of meat breeds such as the Dorset [41] and Sabi [56], at 86% and 88%, respectively, were also lower than dual-purpose breeds like the Corriedale [28,29] and Targhee [45], which had efficiencies of 96% and 97%, respectively. Estimates for the milk breeds were severely limited, possibly due to production systems in which lambs are weaned at a very young age (between 30 and 60 days) or are reared artificially from birth [87,88]. Two examples are the East Friesian cross [37] and Chios cross [27], with efficiencies of 89% and 124%, respectively. Finally, examples of high efficiencies from the prolific group were the Flevolander [21] and Perendale FecB [23], with values of 158% and 152%, respectively.

The data also indicates wide variation in reproductive efficiency at high levels of fecundity. For example, reproductive efficiency can be increased markedly in some flocks to near 180–190% (Figure 3e) by improving fecundity. A specific example is where the FecB gene was inserted in the Rambouillet, which increased efficiency from 75% [41,60] to 186% [20], but this was achieved by applying a high level of management. In contrast, introduction of the FecB gene into the Merino, though managed under extensive conditions, resulted in poor efficiency (77%), mainly resulting from low lamb survival (39%) in a flock with fecundity at 236% [65].

Fertility, as a component of reproductive efficiency, was significantly higher in the dual-purpose breed class (5–6%; *p* < 0.05; Table 2) than either the wool or meat classes. Typical of the dual-purpose breeds, with fertility values near the mean value (87.5%), were the Corriedale [28,29], Romney [32,34,76], Columbia [58], Border Leicester x Merino [28,29] and the Scottish Blackface [69]. Those breeds typical for the wool and meat classes, where means for fertility were 81.3% and 83.2%, respectively (Table 2), were the fine-wool Merino [70], medium-wool Merino [51] and strong-wool Merino [28,29]; Tsigai [46], Dorper [57] and the Suffolk [30] represented the meat class. Higher observed fertility of the dual-purpose compared with the wool (Merino) class is in line with the results of Atkins (1980a,b) [28,29] where differences were 8% and 13% higher for the Corriedale and Border Leicester x Merino, respectively, and where animals were managed under the same environmental conditions.

### 4.3. Within Breed Classes

#### 4.3.1. Wool Groups

There were some interesting variations in reproductive efficiency within breed classes. Fine-wool Merino sheep were least efficient compared with medium- and strong-wool strains, a result consistent with the reports of Mortimer et al. (1985) [89] and Swan et al. (2001) [72]. While reproductive efficiency in fine-wools has a negative genetic correlation with fibre diameter [70], it was interesting that fecundity was primarily associated with reduced efficiency. This finding supports our results for low fecundity in the fine-wool cohort (Table 3) and is the main reason for differences in efficiency between the Merino fine-, medium- and strong-wool strains. Also interesting was that there were no such negative genetic correlations for efficiency when selecting for reduced fibre diameter in medium- and strong-wool strains [90]. Given that fecundity was positively and strongly associated with ovulation rate, it is likely that genetic differences between fine-wool and other strains are controlled by genes altering oocyte recruitment and selection.

#### 4.3.2. Meat Groups

The main feature of the meat class was the higher reproductive efficiency of the narrow-tail group (101%) compared with the fat-tail (82%), shedder (87%) and hair (77%) groups, mainly due to differences in fecundity. Fat tail breeds are mainly found in semi-arid areas such as the Middle East, where nutritional conditions may be more challenging and the ability to raise more than one lamb is difficult [91]. Exceptions are the Assaf and Awassi fat-tail types. The Assaf was developed from an Awassi x East Friesian cross and has moderate fecundity and milk production [92], while the Improved Awassi was selected for high milk production but has low fecundity (130%) [93]. While information on their milk-producing ability is available, measures of their reproductive efficiency (lambs weaned of ewes exposed to rams) has not been reported. Introgression of the Fec gene in both breeds (Afec Assaf and Afec Awassi) has lifted fecundity to 250 and 190%, respectively [94]. High levels of fecundity for these breeds would suggest their potential for high levels of reproductive efficiency, given the right environmental conditions. The narrow-tail class includes breeds such as the Dorset, Suffolk and Texel, which are commonly used as terminal sires to improve meat quality aspects in crossbreeding systems found in countries such as Australia [95] and the United Kingdom [96]. Wool-shedding breeds such as the Dorper, which has low fecundity and efficiency [57] and is suited to the semi-arid areas of the world, have become popular in Australia, particularly in pastoral areas [97] and, to a lesser extent, in higher rainfall areas due to reduced cost of management and sustained meat prices.

#### 4.3.3. Milk Groups

Estimates of reproductive efficiency and reproductive components for milk groups were limited to three reports (Table 3). Two reports were from Turkey, where the Awassi fat-tail [48] and a Chios crossbreed fat-tail [27] were evaluated as potential milk breeds. The third report was an assessment of milk yield and growth of lambs from the East Friesian milk breed crossed with the Corriedale [37] in Uruguay. These studies indicated reproductive efficiencies of 89–96%, with low fecundity (123–125%). However, singular reports for fecundity where milk is the primary end use of the breed indicate that fecundity can be high for the East Friesian [98] (210%), Chios [99] (190%) and [100] (207%), Afec Awassi and Afec Assaf [94] (250% and 190%), and moderately high for the Manchega [101] (146%). This would indicate that potential reproductive efficiencies of these milk breeds could be high given their ability to produce a high litter size.

In extensive to semi-intensive systems employed by milk sheep farmers, as commonly found in northern Mediterranean countries, reproductive performance may be relatively unchallenged since fecundity was found to be at low to moderate levels (126–136%) and fertility was high (97.3–97.5%), as was lamb survival (91.3–89.4%) [102,103]. However, in farming systems using high-milk-producing and highly prolific breeds, such as the East Friesian, Lacaune, Sarda, Chios and Assaf, the demands on reproductive performance of the ewe are elevated. In this system, meat and milk are important contributors to farmer income. Intensive management is required to meet the high energy/protein requirements of the animals. While an increase in fecundity has a small effect on increasing milk yield, higher milk yield may delay conception due to negative energy balance [104], which is also an issue in high-producing dairy cattle [105]. In addition, factors such as a negative correlation of milk yield with fertility [106], increased susceptibility to disease [107], artificial insemination issues [108], metabolic demands caused by pregnancy and lactation [109], higher milk yields and reduced ovulation rates [110], increased vaginal prolapses [111]), the need for hormone-free reproduction management [112] and seasonality [113] are just some of the issues that impact reproductive efficiency in highly productive milk- and meat-producing animals.

#### 4.3.4. Prolific Groups

Reproductive efficiency may be higher for those prolific sheep where ovulation is controlled by many genes (Romanov; Akat and Cam, 2024 [25] and Wolfova et al., 2011 [114]; D’man; Boujenane et al., 2013 [19]; Finn composites; Flevolander-van Haandel and Visscher, 1995 [21] and ABRO-Martin et al., 1981 [31]; Finn purebred; Fogarty et al., 1984 [41] compared with prolific sheep introgressed with the FecB gene (Merino; Kleemann and Walker, 2005a [65]; Rambouillet; Southey et al., 2002 [20]; Romney and Perendale; Meyer et al., 1994 [23]. The numerical difference in efficiency (153% vs. 125%; Table 3) was mainly due to higher lamb survival (79% versus 55%) in the prolific group, where ovulation is controlled by many genes. To maintain high reproductive efficiency in sheep introgressed with the FecB gene, it is necessary to choose base breeds with inherently good rearing ability, such as the Romney and Perendale [23], or a composite of breeds, such as the Suffolk, Dorset and Border Leicester (Earl, Colin; pers comm). While prolific breeds are normally managed under semi-intensive and intensive production systems [12], their performance has been tested under extensive grazing conditions [65]. Whether the difference in reproductive efficiency between single- and multiple-gene flocks reported in this study was due to genetic or management effects remains to be determined.

Other prolific breeds, where ovulation is controlled by many genes and which have moderate to high fecundity and survival (suggesting they would have high reproductive efficiency), are the Katahdin, Barbados Blackbelly, Leyn and Polypay [115,116,117,118]). These breeds, along with others of known high fecundity, (Arcott, Chios, Swifter and Zwarbles, Mule, iLe de France and Blanche du Massif Central, Cambridge, Small-tail Han and Hu [119,120,121,122,123,124,125,126]) are also candidates for high reproductive efficiency.

### 4.4. Specific Breeds

While it is interesting to note that breeds with the highest and lowest efficiencies were the D’man and Santa Ines x Crioula, respectively (192% versus 19%), they were estimated with high standard errors (Table 4). Nevertheless, listing the performance of specific breeds for reproductive characteristics informs researchers, specialist breeders and commercial growers of their relative merit. Breeds that provided robust estimates of reproductive efficiencies were the Merino (fine, medium, strong; 74.4 ± 4.58, 90.6 ± 3.71, 88.3 ± 11.49; *n* = 5, 13, 4; respectively), Romney (113.4 ± 5.20, *n* = 5), Corriedale (106.1 ± 11.40, *n* = 4), Targhee (97.3 ± 5.15, *n* = 4), Rambouillet (83.2 ± 9.00, *n* = 4) and Dorper (86.6. ± 4.93, *n* = 3).

### 4.5. Country

Estimates of reproductive efficiency, though not very robust for many countries, do highlight the issues that confront countries where estimates are sound, such as for Australia, New Zealand, the U.S., South Africa and Turkey. Values for fertility, fecundity and lamb survival were all greater for sheep in New Zealand compared to Australia. It could be argued that differences between the countries were mainly due to the varied performance of the dominant breeds in the two countries—the Romney and Merino, respectively—and possibly due to variations in environmental conditions. Areas for improving reproductive efficiency in the Romney were identified from surveys of many flocks in New Zealand [33,34], indicating that worthwhile gains in efficiency could be achieved by attending to factors controlling fertility, fecundity and lamb survival. Similar large-scale surveys were conducted on the Merino in Australia [65,80,81,127], which benchmarked reproductive efficiency and its components during the latter decades of the 20^th^ century. Lindsay et al. (1975) [81] highlighted the need to improve fecundity (via stimulation of the ovulation rate) and fertility, while the studies that followed [65,80,127,128,129] mainly focussed attention on improvement of lamb survival. Since then, substantial efforts by sheep industry members (represented by Meat and Livestock Australia Ltd., and Australian Wool Innovation) in concert with research [130,131,132,133,134,135,136,137,138,139,140,141,142,143,144] and advisory groups have lifted reproductive efficiency of the Australian flock by approximately 10% [145]. This was achieved by attending to both genetic and environmental means of improving all reproductive components with emphasis on lamb survival. In comparison to Australia’s average lamb marking percentage during 2010–2020 of 98%, New Zealand [146] and the U.S. [147] lamb crops averaged 124% and 106%, respectively. These figures for Australia, New Zealand and the U.S. are above those given in the current study (Table 5) and are likely to indicate progress made by these countries in improving reproductive efficiency in recent decades.

### 4.6. World Regions

Flocks in European countries were the most efficient reproductively (110%) of all world regions due to high fecundity (154%) and lamb survival (87%; Table 6). This result contrasts with the North American region, where fecundity was also high (143%) but survival of lambs was the lowest of all world regions (73%), lowering reproductive efficiency to 90%. In the latter world region, poor survival (<70%) was found in Columbia [58], Targhee [45,148], and Rambouillet [41] breeds, as well as in Finn flocks (<60%) [41]. In comparison, other regions, such as the Middle East/North Africa, and East Asia/Western Pacific, had flocks of lower fecundity (129% and 130%, respectively) but with reasonable lamb survival (84% and 81%, respectively) and reproductive efficiency (95% and 88%, respectively). Also of note was the similarity of fertility between world regions, which was generally more than 80%.

Representation of the world’s regional sheep population by the current data was poor. For example, China has the largest sheep population in the world (193 m of 1.3 b; [2], yet was not represented in the current data for the East Asia and Pacific region. In contrast, Australia and New Zealand, which also have large flocks (72 m and 29 m, respectively) and are included in the same region as China, are well represented in the current data with a plethora of reports (40 and 14, respectively; Table 5). Other notable anomalies are the very low representation of flocks in the Sub-Saharan African region, with one of the world’s largest sheep populations (367 m) [2] but only six records available, and the relatively high representation of records from North America (*n* = 24), a region that makes the lowest contribution to the world’s sheep population (17.5 m of 1.3 b) [2]. Even within a region such as Europe, a disproportionate number of records were provided by a few countries (Turkey, Hungary, Romania; 16 out of 20 records).

### 4.7. Practical Implications

There are number of practical implications that may result from the findings of this study. One example is a climate change scenario in which it is anticipated that increased temperatures may impact reproductive performance. The relationships and mean data established for reproductive components by this study have allowed prediction of the climate’s influence on reproductive efficiency. It is known that as the number of days per week with ambient temperatures ≥32 °C degrees increases during the summer mating period, fertility declines. A relationship for this effect was established by Kleemann et al. (2005a) [80]. Utilising this relationship to calculate the effect of increasing temperature by an extra day per week, and applying an exponential equation relating fertility with reproductive efficiency (current study; Figure 2d) and the mean value for global fertility (current study; Table 1), it was calculated that the number of lambs weaned per 100 ewes exposed to rams fell from 93.9% to 88.7%. If it is assumed that 60% of the world’s sheep population of 1.3 billion are breeding ewes and that the fertility of 50% of these is affected by increased exposure to higher temperatures, then it is predicted that there would be 20.3 million fewer lambs at weaning annually. This is only one example of many where the relationships established from this study could be applied for predicting reproductive outcomes.

### 4.8. Limitations of the Study and Future Research

Although the dataset represents only a small proportion of breeds that exist in the world, estimated to be <5%, there was a diversity of breed types (fat-tail, hair, wool-shedders, prolific and milk types, as well as the more prevalent breeds grown for meat and wool), that contributed to the dataset. In addition, the study examined a diversity of breeds where the animals were managed in countries scattered globally. Furthermore, the study was limited by the availability of information from non-English speaking countries, such as China and those in Sub-Saharan Africa (both of which have substantial populations of sheep) and by the eligibility criteria that all three reproductive components be available to estimate reproductive efficiency. As a result, country effects should be interpreted cautiously, as they may reflect the clustering of research efforts and production systems rather than fully independent national differences. The two limitations of low breed and country/region representation indicate that caution be exercised when referring to the current study as providing true globally relevant information. To address these limitations, it is suggested that future studies benchmark the reproductive performance of sheep in China and Sub-Saharan Africa, where there are large populations of fat-tail and thin-tail types [149,150]. The current study indicated that fat-tail breeds have low fecundity and reproductive efficiency, but this needs to be confirmed for these regions. Such studies would not only focus research and advisory activities for improving reproductive efficiency but also provide much needed information for improving global prediction models, such as those of the current study.

Future research could assess the impact that new technologies, such as those associated with artificial reproductive protocols, have on improving components of the reproductive process, and that may increase reproductive efficiency. Closer examination of the relationship between fecundity and fertility from the global literature is likely to yield useful insights into the biological reasons for the small but positive association. This could be achieved by the collection of a larger dataset than was possible for the current study, where eligibility criteria required all three reproductive components to be present. Another area of research worth considering relates to the resilience of breeds, such as the fat-tails, wool-shedders and hair type sheep, to climate change and its effect on their components of reproduction and efficiency. Adaptations of the fat-tail to poor environmental conditions—such as slow release of fat from the tail for energy demand, an attribute that allows the animal to survive over extensive periods, as well as modification of the lipid metabolite mechanism that limits the toxic effects of ketones—are important features of this breed group [151]. Although fat-tails breeds may have low reproductive efficiency, primarily due to low fecundity, they have the potential in a breeding program to generate animals where the important adaptations mentioned above are maintained while selecting for increased fecundity and reproductive efficiency. A good example of this approach has been demonstrated in the Barbarine fat-tail [152].

## 5. Summary and Conclusions

Reproductive efficiency of the world’s sheep population was estimated at 97.7 lambs weaned per 100 ewes exposed to rams, with a range of 19–192%. This estimate was derived from only 51 breeds or breed crosses compared with approximately 1114 breeds known to exist in the world (<5%). Values for fertility and lamb survival, two components of efficiency, were low in value (84% and 81%, respectively) while the third component, fecundity, was of moderate size (145%). Relationships between these components indicated no association between fecundity and fertility R^2^ = 0.003, *p* > 0.05), a positive relationship between lamb survival and fertility (R^2^ = 0.226, *p* < 0.001) and a negative relationship for survival and fecundity (R^2^ = 0.176, *p* < 0.001). Efficiency increased at a decreasing rate with increasing fecundity (R^2^ = 0.490, *p* < 0.001), such that maximum efficiency reached about 150% when fecundity was at 250%. Relationships of components examined within breed classes were similar except for the meat and prolific classes, where lamb survival declined at a greater rate than other classes as fertility declined (R^2^ = 0.8265, *p* < 0.001 and R^2^ = 0.2078, *p* < 0.1, respectively). Within breed classes, medium- and strong-wool Merinos were more efficient than the fine-wool, while narrow-tailed meat breeds were more efficient than the fat-tail, wool-shedders and hair types. Limited data for the milk breeds indicated low to moderate efficiency that was associated with low fecundity of the breeds studied. Reproductive efficiency appeared to be higher in prolific breeds where fecundity (ovulation) was controlled by many genes compared with those breeds with a single gene, such as FecB. Specific breeds with the most robust estimates (low standard errors) were the Merino, Romney, Corriedale, Targhee, Rambouillet and Dorper. Benchmarking of reproductive efficiency of sheep breeds in various countries was best demonstrated by studies in New Zealand and Australia, where examination of reproductive components enabled the sheep industry to focus research and educational effort on those components needing the most improvement. Examination of reproductive efficiency within world regions highlighted the disparity between regions with the largest sheep populations and the lack of information describing how well sheep perform reproductively. While the current data for reproductive efficiency and its components was derived from the world’s scientific literature, irrespective of breed and the impact of environmental factors, there is a need to further define reproductive efficiency and establish its relationships with major factors such as age-of-ewe, nutrition, season and climate.

In conclusion, this article provides an overview of the reproductive efficiency of the world’s sheep population (though based on <5% of breeds), defines and quantifies how the parameters controlling efficiency interact, and estimates contributions made by breed, country and world region toward reproductive output.

## Figures and Tables

**Table 1:** Descriptive statistics for fertility (ewes pregnant per 100 ewes exposed to rams), fecundity (lambs born of ewes pregnant), lamb survival (lambs present at weaning of lambs born) and lambs weaned (lambs weaned of ewes exposed to rams) and number of experiments where all reproductive components were recorded.

Reproductive Component	*n* ^1^	Mean	SE	SD	Minimum	Maximum
Fertility	112	83.86	0.95	10.09	42	98
Fecundity	112	144.58	3.48	36.92	102	268
Lamb survival	112	80.77	0.93	9.84	38	100
Lambs weaned	112	97.71	2.68	28.50	19	192
^2^ Number of animals within experiments		4525.03			25	42,831

^1^ Number of experiments (*n*) = number of means sourced from scientific papers; ^2^ Number of animals = number contributing to reproductive component mean values given in scientific papers.

**Table 2:** Weighted means ± SEM (*n*) for fertility (ewes pregnant per 100 ewes exposed to rams), fecundity (lambs born of ewes pregnant), lamb survival (lambs present at weaning of lambs born) and lambs weaned (lambs weaned of ewes exposed to rams) of breeds categorised according to their main use (wool, dual-purpose, meat, milk, prolific).

Breed Class	*n*	Fertility	Fecundity	Lamb Survival	Lambs Weaned
Wool	34	81.3 ± 0.96 ^a^	126.4 ± 2.15 ^a^	79.7 ± 0.89 ^a^	81.5 ± 2.20 ^a^
Dual-purpose	39	87.5 ± 1.07 ^b^	139.5 ± 2.37 ^b^	78.3 ± 0.99 ^a^	95.7 ± 2.43 ^b^
Meat	25	81.6 ± 1.21 ^a^	122.0 ± 2.50 ^a^	87.0 ± 1.04 ^b^	86.8 ± 2.56 ^ab^
Milk	3	85.9 ± 21.63 ^ab^	124.3 ± 48.13 ^abc^	86.3 ± 20.05 ^ab^	91.8 ± 49.31 ^abc^
Prolific	12	82.2 ± 4.33 ^ab^	238.1 ± 9.64 ^c^	77.1 ± 4.01 ^ab^	149.8 ± 9.87 ^c^

Within columns, means with different superscript letters are significantly different (*p* ≤ 0.05).

**Table 3:** Weighted means ± SEM (*n*) for fertility (ewes pregnant of 100 ewes exposed to rams), fecundity lambs born of ewes pregnant), lamb survival (lambs present at weaning of lambs born) and lambs weaned (lambs weaned of ewes exposed to rams) of breeds categorised within their main use (wool—fine, medium, strong, unknown; meat—narrow-tail, fat-tail, shedder, hair; milk—narrow-tail, fat-tail; prolific—many genes, Fec gene).

Breed Group	*N*	Fertility	Fecundity	Lamb Survival	Lambs Weaned
Wool (fine)	5	79.8 ± 2.04 ^a^	115.1 ± 4.20 ^a^	81.5 ± 1.91 ^a^	74.4 ± 4.55 ^a^
Wool (medium)	13	83.0 ± 1.65 ^a^	138.5 ± 3.40 ^b^	79.5 ± 1.55 ^a^	90.6 ± 3.69 ^a^
Wool (strong)	4	81.2 ± 5.12 ^a^	135.1 ± 10.55 ^ab^	79.3 ± 4.80 ^a^	88.3 ± 11.43 ^a^
Wool (no specification)	12	80.6 ± 1.49 ^a^	121.8 ± 3.07 ^ab^	78.8 ± 1.40 ^a^	77.4 ± 3.33 ^a^
Meat	12	89.9 ± 4.17 ^a^	134.2 ± 8.60 ^a^	83.9 ± 3.91 ^a^	101.3 ± 9.31 ^a^
Meat (fat-tail)	6	86.4 ± 3.10 ^a^	110.7 ± 6.40 ^a^	85.3 ± 2.91 ^a^	82.3 ± 6.93 ^a^
Meat (shedder)	4	80.0 ± 1.27 ^a^	123.0 ± 2.62 ^a^	88.0 ± 1.19 ^a^	86.6 ± 2.83 ^a^
Meat (hair)	3	81.0 ± 5.92 ^a^	117.2 ± 12.20 ^a^	78.8 ± 5.55 ^a^	76.7 ± 13.21 ^a^
Milk (fat-tail)	2	89.5 ± 34.68	123.1 ± 71.52	86.8 ± 32.55	95.9 ± 77.47
Prolific (many genes)	7	81.3 ± 4.53 ^a^	238.7 ± 9.34 ^a^	79.2 ± 4.25 ^a^	152.9 ± 10.11 ^a^
Prolific (Fec gene)	5	89.9 ± 13.37 ^a^	233.5 ± 27.56 ^a^	59.3 ± 12.54 ^a^	122.9 ± 29.86 ^a^

Within columns and within end use categories means with different letters are significantly different (*p* < 0.05). Those means with different upper-case letters tend to be significant (*p* < 0.1).

**Table 4:** Weighted means ± SEM (*n*) for fertility (ewes pregnant of 100 ewes exposed to rams), fecundity (lambs born of ewes pregnant), lamb survival (lambs present at weaning of lambs born) and lambs weaned (lambs weaned of ewes exposed to rams) categorised according to breed when all reproductive components are present. Reproductive efficiency (lambs weaned) is listed in descending order.

Breed (Reference Number)	*n*	Fertility	Fecundity	Lamb Survival	Lambs Weaned
D’man [19]	1	94.4 ± 9.11	238.0 ± 19.05	85.3 ± 5.72	191.6 ± 19.10
Rambouillet FecB+ [20]	1	92.6 ± 50.01	266.0 ± 104.58	75.4 ± 31.38	185.7 ±104.83
Flevolander [21]	1	72.8 ± 7.68	268.0 ± 16.05	81.0 ± 4.82	158.0 ± 16.09
Turcan x Blueface Leicester [22]	2	95.7 ± 38.18	171.9 ± 79.84	94.2 ± 23.96	155.3 ± 80.03
Perendale FecB+ [23]	1	94.0 ± 27.87	260.0 ± 58.27	62.3 ± 17.49	152.3 ± 58.41
Rambouillet Fec++ [20]	1	96.0 ± 80.14	161.0 ± 167.57	95.1 ± 50.29	147.0 ± 167.97
Romney FecB+ [23]	1	93.0 ± 34.91	254.0 ± 72.99	62.2 ± 21.91	146.9 ± 73.17
Tazegzawt [24]	1	87.0 ± 59.55	182.9 ± 124.53	88.0 ± 37.37	140.0 ± 124.82
Romanov [25]	1	88.2 ± 35.74	176.0 ± 74.74	88.6 ± 22.43	137.6 ± 74.92
Bonga [26]	1	95.0 ± 19.12	145.0 ± 39.98	95.0 ± 12.00	131.0 ± 40.08
Chios x Kivircik [27]	1	88.0 ± 97.50	141.0 ± 203.86	100.0 ± 61.18	124.1 ± 204.35
Border Leicester x Merino [28,29]	1	92.1 ± 20.26	166.0 ± 42.36	79.6 ± 12.71	121.7 ± 42.46
Targhee x Suffolk [30]	2	84.6 ± 34.56	171.8 ± 72.26	83.6 ± 21.68	121.1 ± 72.43
ABRO Finn synthetic [31]	1	86.0 ± 11.64	169.0 ± 24.33	81.1 ± 7.30	117.9 ± 24.39
Perendale Fec++ [23]	1	94.0 ± 30.65	143.0 ± 64.08	87.4 ± 19.23	117.5 ± 64.24
Suffolk x Targhee [30]	2	89.9 ± 27.47	151.7 ± 57.43	84.5 ± 17.24	115.0 ± 57.57
Romney [23,32,33,34,35]	5	93.6 ± 2.48	136.0 ± 5.19	88.7 ± 1.56	113.4 ± 5.20
Romney Fec++ [23]	1	93.0 ± 37.29	140.0 ± 77.95	87.1 ± 23.39	113.4 ± 78.13
Perendale [23]	1	91.0 ± 18.08	147.0 ± 37.80	84.4 ± 11.35	112.9 ± 37.89
Corriedale [28,29,36,37,38]	4	91.2 ± 5.44	136.3 ± 11.37	84.2 ± 3.41	106.1 ± 11.40
Turcan [22]	2	95.9 ± 39.67	119.2 ± 82.95	92.3 ± 24.89	105.7 ± 83.15
DLS Composite [39]	1	86.3 ± 39.80	144.0 ± 83.23	83.5 ± 24.98	103.8 ± 83.42
Dorset [40,41]	2	90.7 ± 4.73	132.3 ± 9.89	84.1 ± 2.97	101.1 ± 9.92
Akkaraman x Merino [42]	1	92.2 ± 43.09	127.1 ± 90.09	85.3 ± 27.04	100 ± 90.31
Finn [39,41]	3	71.2 ± 13.74	252.5 ± 28.74	55.2 ± 8.62	99.2 ± 28.81
Hyfer [43]	1	79.3 ± 12.12	146.0 ± 25.33	85.7 ± 7.60	99.2 ± 25.39
Maternal Australia [44]	1	68.0 ± 19.52	176.0 ± 40.80	81.0 ± 12.25	99.0 ± 40.90
Targhee [30,41,45]	4	87.8 ± 2.56	150.1 ± 5.14	73.6 ± 1.54	97.3 ± 5.15
Tsogai [46]	1	82.8 ± 26.06	145.0 ± 54.48	79.6 ± 16.35	95.6 ± 54.61
Lori-Bakhtiari [47]	1	90.0 ± 6.65	117.0 ± 13.91	89.3 ± 4.17	94.0 ± 13.94
Romney x Border Leicester [32]	4	88.2 ± 7.24	127.9 ± 15.13	82.8 ± 4.54	93.6 ± 15.17
Suffolk [30,41]	3	77.4 ± 22.75	160.3 ± 47.58	75.0 ± 14.28	93.6 ± 47.69
Kivircik [27]	1	75.0 ± 92.12	133.0 ± 192.63	92.9 ± 57.81	92.6 ±193.09
Awassi [48]	1	89.8 ± 40.21	120.0 ± 84.07	84.5 ± 25.23	91.1 ± 84.27
Makooei [49]	1	93.0 ± 8.34	116.0 ± 17.44	84.5 ± 5.23	91.1 ± 17.48
Merino medium [28,29,50,51,52,53,54,55]	13	83.0 ± 1.77	138.5 ± 3.70	79.6 ± 1.11	90.6 ± 3.71
East Friesian x Corriedale [37]	1	83.6 ± 29.18	125.0 ± 61.02	86.1 ± 18.31	89.2 ± 61.17
Sabi [56]	1	88.0 ± 7.75	116.0 ± 16.20	87.0 ± 4.86	88.8 ± 16.24
Merino strong [28,29,52,53]	4	81.2 ± 5.48	135.1 ± 11.46	79.3 ± 3.44	88.3 ± 11.49
Akkaraman [42]	1	91.7 ± 8.49	102.4 ± 17.75	93.5 ± 5.33	87.8 ± 17.79
Dorper [46,57]	3	80.0 ± 2.35	123.1 ± 4.91	88.0 ± 1.48	86.6 ± 4.93
Columbia [58]	2	86.0 ± 1.95	138.5 ± 4.07	71.2 ± 1.22	84.8 ± 4.07
Rambouillet [41,59,60]	4	77.3 ± 4.36	127.7 ± 9.12	84.4 ± 2.74	83.2 ± 9.14
Merino various [27,38,42,44,52,61,62,63,64,65,66,67,68]	13	80.8 ±1.53	124.6 ± 3.21	79.2 ± 0.96	79.7 ± 3.21
Scottish Blackface [69]	1	85.8 ± 6.51	109.4 ± 13.61	83.7 ± 4.08	78.8 ± 13.64
Merino FecB+ [65]	1	83.2 ± 24.65	235.5 ± 51.55	39.4 ±15.47	76.9 ± 51.67
Polwarth [28,29,38]	2	87.2 ± 12.37	114.7 ± 25.87	76.2 ± 7.77	75.7 ± 25.94
Merino fine [50,52,70,71,72]	5	79.8 ± 2.18	115.1 ± 4.57	81.5 ± 1.37	74.4 ± 4.58
Afar [73]	1	90.0 ± 23.79	106.0 ± 49.74	75.0 ± 14.93	72.0 ± 49.86
Barki [74]	1	79.0 ± 5.23	104.7 ± 10.94	79.2 ± 3.28	65.5 ± 10.96
Crioula [75]	1	75.0 ± 12.70	120.0 ± 26.55	70.0 ± 7.97	63.0 ± 26.61
Santa Ines x Crioula [75]	1	42.0 ± 22.25	119.0 ± 46.52	38.0 ±13.96	19.0 ± 46.64

**Table 5:** Weighted means ± SEM (*n*) for fertility (ewes pregnant of 100 ewes exposed to rams), fecundity (lambs born of ewes pregnant), lamb survival (lambs present at weaning of lambs born) and lambs weaned (lambs weaned of ewes exposed to rams) of breeds categorised according to country.

Country	Reference Number	*n*	Fertility	Fecundity	Lamb Survival	Lambs Weaned
Algeria	[24]	1	87.0 ± 52.97	182.9 ± 141.79	88.0 ± 38.49	140.0 ± 118.10
Australia ^1^	see footnote	40	82.1 ± 0.88 ^a^	128.2 ± 2.35 ^a^	79.9 ± 0.64 ^a^	83.9 ± 1.96 ^a^
Brazil	[75]	2	66.9 ± 9.81 ^ab^	119.8 ± 26.26 ^ab^	62.2 ± 7.13 ^a^	52.2 ± 21.87 ^ab^
Canada	[39]	2	89.1 ± 29.92 ^ab^	160.9 ± 80.09 ^ab^	83.8 ± 21.74 ^abc^	121.2 ± 66.71 ^ab^
Egypt	[74]	1	79.0 ± 4.65	104.7 ± 12.45	79.2 ± 3.38	65.5 ± 10.37
Ethiopia	[73]	1	95.0 ± 17.01	145.0 ± 45.52	95.0 ± 12.36	131.0 ± 37.92
Hungary	[46]	3	82.8 ± 18.68 ^ab^	141.3 ± 49.99 ^ab^	81.1 ± 13.57 ^abc^	94.7 ± 41.64 ^ab^
Iran	[47,49]	2	91.2 ± 4.63 ^ab^	116.6 ± 12.38 ^ab^	87.4 ± 3.36 ^ab^	92.9 ± 10.31 ^ab^
Morocco	[19]	1	94.4 ± 8.10	238.0 ± 21.69	85.3 ± 5.89	191.6 ± 18.07
The Netherlands	[21]	1	72.8 ± 6.83	268.0 ± 18.28	81.0 ± 4.96	158.0 ± 15.22
New Zealand	[23,32,33,34,35,76]	14	93.0 ± 2.05 ^bA^	136.8 ± 5.48 ^ab^	87.7 ± 1.49 ^b^	111.8 ± 4.57 ^b^
Romania	[22]	4	95.8 ± 24.47 ^ab^	146.6 ± 65.50 ^ab^	93.3 ± 17.78 ^abc^	131.5 ± 54.55 ^ab^
South Africa	[57,62]	4	80.0 ± 1.20 ^a^	123.1 ± 3.21 ^a^	87.9 ± 0.87 ^b^	86.6 ± 2.67 ^a^
Turkey	[25,27,42,48,63]	9	91.1± 5.00 ^ab^	124.3 ± 13.39 ^ab^	94.0 ± 3.64 ^b^	106.3 ± 11.16 ^ab^
UK	[31,69]	2	85.8 ± 5.05 ^ab^	123.6 ± 13.52 ^ab^	83.1 ± 3.67 ^abc^	88.1± 11.27 ^ab^
Uruguay	[37,71]	2	74.3 ± 5.16 ^abB^	123.8 ± 13.80 ^ab^	78.4 ± 3.75 ^abc^	72.0 ± 11.50 ^ab^
USA	[20,30,37,41,45,58,60]	22	85.4 ± 1.27 ^ab^	142.8 ± 3.39 ^b^	73.3 ± 0.92 ^c^	89.2 ± 2.82 ^a^
Zimbabwe	[56]	1	88.0 ± 6.89	116.0 ± 18.45	87.0 ± 5.01	88.8 ± 15.37

Within columns, means with different lower-case superscript letters are significantly different (*p* ≤ 0.05). Those means with different upper-case letters tend to be significant (*p* < 0.1). ^1^ Reference numbers for Australia: [29,36,38,40,43,44,50,51,52,53,55,61,64,65,66,67,68,70,72].

**Table 6:** Weighted means ± SEM (*n*) for fertility (ewes pregnant of 100 ewes exposed to rams), fecundity (lambs born of ewes pregnant), lamb survival (lambs present at weaning of lambs born) and lambs weaned (lambs weaned of ewes exposed to rams) of breeds categorised according to world region.

World Region	*n*	Fertility	Fecundity	Lamb Survival	Lambs Weaned
EurCAsia	19	85.3 ± 3.42 ^a^	154.2 ± 10.63 ^a^	86.9 ± 2.51 ^ac^	110.2 ± 8.94 ^a^
MENAfr	5	86.4 ± 3.36 ^a^	128.8 ± 10.46 ^a^	83.6 ± 2.47 ^ac^	95.2 ± 8.80 ^a^
NAm	24	85.4 ± 1.40 ^a^	142.8 ± 4.36 ^a^	73.3 ± 1.03 ^b^	89.3 ± 3.66 ^a^
EAsiaPac	54	83.8 ± 0.89 ^a^	129.5 ± 2.78 ^a^	81.1 ± 0.66 ^a^	88.2 ± 2.34 ^a^
SAm	5	73.0 ± 4.98 ^a^	123.0 ± 15.50 ^a^	75.2 ± 3.65 ^ab^	68.4 ± 13.03 ^a^
SSuharAfr	5	81.0 ± 2.14 ^a^	122.7 ± 6.06 ^a^	87.6 ± 1.57 ^ac^	87.1 ± 5.60 ^a^
SAsia	0	-	-	-	-

World regions: Europe and Central Asia (EurCAsia); Middle East and North Africa (MeNAfr); North America including central America and Greenland (NAm); East Asia and Pacific (EAPac); South America (SAm); Sub-Saharan Africa (SSuharAfr); India, Pakistan, Afghanistan (South Asia). Within columns, means with different superscript letters are significantly different (*p* ≤ 0.05).

**Table 7:** Summary of random effects meta-regression results for reproductive traits.

Trait	Outcome Type	k	I^2^ (%)	R^2^ (%)	Key Significant Moderators
Fertility	Proportion	112	99.6	20.9	World region
Fecundity	Continuous	112	99.9	74.9	Year, world region, breed class
Survival	Proportion	112	99.5	41	World region, breed class
Lambs weaned	Continuous	112	100	41.8	Year, world region, breed class

## Data Availability

Details of the dataset can be supplied under reasonable request. The articles sourced to build the dataset are supplied in the Supplementary bibliography.

## Supplementary Materials

The following supporting information can be downloaded at: https://www.mdpi.com/article/10.3390/ani16111608/s1. File S1: PRISMA 2020 Checklist. Table S1: Protocol for systematic review. Table S2: Eligibility criteria. Table S3: Full database search strategies. Table S4: PRISMA flow counts. Table S5: Risk of bias assessment (SYRCLE). Table S6a: Sensitivity analyses: omnibus test statistics and variance (R^2^) for reproductive components. Table S6b: Sensitivity analyses: estimated marginal means (±SE) for each breed class under both weighting schemes. Table S7: Weighted means ± SEM (*n*) for fertility, fecundity, lamb survival and lambs weaned categorised according to breed. Figure S1: Weighted means ± SEM for fertility, fecundity, lamb survival and lambs weaned within breed class. Figure S2. Weighted means ± SEM for fertility, fecundity, lamb survival and lambs weaned for breed class and group. Figure S3: Weighted means ± SEM for fertility, fecundity, lamb survival and lambs weaned for breed class and group (wool—fine, medium, strong, unknown; meat—narrow-tail, fat-tail, shedder, hair; milk—narrow-tail, fat-tail; prolific—many genes, Fec gene). Figure S4: Weighted means ± SEM for fertility, fecundity, lamb survival and lambs weaned of breeds categorised by country. Figure S5: Weighted means ± SEM for fertility, fecundity, lamb survival and lambs weaned of breeds categorised by world region.

## Abbreviations

The following abbreviations are used in this manuscript:

FecB	Booroola fecundity gene
PRISMA	Preferred Reporting Items for Systematic Reviews and Meta-Analysis
SYRCLE	SYstematic Review Centre for Laboratory animal Experimentation
