# Supplementary material for: Reproductive Efficiency in Sheep: Estimates and Relationships with Fertility, Fecundity and Lamb Survival
Source: Animals (Basel). 2026 May 25;16(11):1608. doi: 10.3390/ani16111608 (PMC13255773; doi:10.3390/ani16111608)
Supplement: Supplementary file 1 [file animals-16-01608-s001.zip › animals-4278450-supplementary.pdf]

Supplementary Tables

Table S1. Protocol for systematic review.

Objectives
To evaluate reproductive efficiency in sheep from global data
Eligibility criteria
Concentrate literature search on large scale studies that are usually conducted to obtain genetic parameters.
Report associated reproductive components (fertility, fecundity, lamb survival (or at least two components if data for reproductive efficiency is available).
Examine mature age ewes but include maiden ewes if mated when 1-2 years of age.
Mating system is performed annually
Search Strategy
Fogarty (1995) and Safari and Fogarty (2003) have scanned the world literature for genetic parameters including those associated with reproduction in sheep. Their literature searches will be the basis for selecting suitable articles published up to and including 2003. An additional search using Web of Science will be employed to find potential articles published from 2004 until late in 2024. A preliminary search using terms such as pregnancy rate, fertility, fecundity, prolificacy, lamb survival yielded more than 800,000 articles. Articles of value that provide robust mean values for the parameters of interest were likely to be produced from genetic studies that require many observations to obtain meaningful estimates of genetic parameters. It was on this basis that the authors will limit the number of articles for initial selection. In addition, the authors are aware of a few studies derived from large datasets that could be considered for selection.
Study selection procedures
Selection procedures for suitable articles are to exclude those that are irrelevant, missing relevant data and contain datasets that are duplicated.
Data extraction items
In addition to articles having relevant and complete data for reproduction other aspects that will be recorded are the number of animals contributing to the means, breed of sheep, breed class (for example, fine wool, medium wool, gene expression of fertility, fat tail, wool shedder), year of publication, and country where the research was conducted.
Planned analysis
Initial analysis of the overall dataset will quantify the means, standard error, range and number of observations for each of the reproductive components and including reproductive efficiency using descriptive statistics in SPSS. The second aim of the analysis will be to examine the relationships between the components and the relationships of each of the components with reproductive efficiency. The third analysis will be to see if the relationships examined above change when examined within breed class. Finally, analysis of variance will be employed to determine any differences between and within breed classes, as well as between countries and between world regions. Analysis will be conducted where the data will be weighted by the number of observations used to determine individual mean values.

Table S2. Eligibility criteria.

Domain	Inclusion	Exclusion
Population	Ewes (Ovis aries), any breed/cross; mature-age means preferred; records from ewes first mated after maturity (1–2 years) included	Non-ovine species; ram- or lamb-only outcomes
Outcomes	Studies reporting means for fertility, fecundity and lamb survival; reproductive efficiency computed/recorded	Studies lacking at least three reproductive component means
Design	Peer-reviewed experimental or observational studies (flock/group means)	Reviews, concept papers, modelling without primary data; conference abstracts without extractable data
Setting	Annual or accelerated lambing systems eligible	—
Special rules	n < 50 allowed only where needed to represent otherwise unrepresented breeds/genotypes; missing components derived from others when feasible	—
Language	English and English translated.	Non-English (acknowledged as potential source of bias)

Table S3. Full database search strategies.

Website of Science Core Collection
Preliminary session as detailed below yielded > 800,000 articles.
Query (paste exactly as run):
TS=(sheep OR ovine OR ewe*) AND TS=(fertility OR "conception rate" OR "pregnancy rate" OR fecundity OR prolificacy OR "litter size" OR "lamb survival" OR "lamb mortality" OR "weaning rate" OR "lambs at weaning") NOT TS=(goat* OR bovine OR cattle) Refined by: Document Types=(ARTICLE); Languages=(ENGLISH) Timespan: 1940–[YYYY] Indexes: SCI-EXPANDED, SSCI, ESCI
Final session
Search
sheep (Topic) and genetic parameters (Topic) and reproduction (Topic) and Article (Document Type)
1:12 PM	Timespan: 2004-01-01 to 2024-11-30 (Index Date)
Web of Science Core Collection

Table S4. PRISMA flow counts.

Stage	Count	Notes
Records identified via database searching	322	From Web of Science
Additional records identified through other sources	18	Author-known studies (n=18);
Additional	92	Review Articles (Fogarty 1995; Safari and Fogarty 2003) containing genetic parameters for reproduction traits
Records after screening (species)	284	Provide count after species screening (Web of Science)
Records screened (title/abstract)	202	Articles unsuitable lacking data on reproductive components (Web of Science)
Full-text articles assessed for eligibility	192	Articles contained reproductive components for young and mature aged ewes (Web of Science n=82; Review source n= 92: Author known source n= 18),
Full-text articles excluded, with reasons	134	Articles did not contain three reproductive components (Web of Science n= 61; Review n=64; Author n=9)
Included in quantitative synthesis	58	Studies reporting all 3 reproductive components, yielding 112 records

Reason for exclusion	Number excluded
Wrong population/species	38
Wrong parameters measured	161
Not all reproductive components measured	134
Review/theoretical/no primary data	41

Table S5. Risk of bias assessment (SYRCLE).

Reference	Study ID	RS	AC	BS	BC	BO	IO	SR	OB	OJ
Turner and Dolling (1965)	10	l	l	l	u	u	l	l	l	l
Mullaney and Hyland (1967)	15	l	u	u	u	u	l	l	l	u
Shelton and Menzies (1968)	20	l	l	u	u	u	l	l	l	u
Hight and Jury (1970a)	21	l	l	l	u	u	l	l	l	l
Hight and Jury (1970b)	22	l	l	l	u	u	l	l	l	l
Shelton and Menzies (1970)	25	l	l	u	u	u	l	l	l	u
Knight et al (1975)	29	h	h	h	u	u	l	u	l	u
Cutten et al (1976)	30	l	l	l	u	u	u	l	l	u
Atkins (1980a)	43	l	l	u	u	u	l	l	l	u
Atkins (1980b)	44	l	l	u	u	u	l	l	l	u
Martin et al (1981)	48	l	l	u	u	u	l	l	l	u
Kelly (1982)	49	h	h	h	u	u	l	l	l	u
Knight et al (1982)	50	h	h	h	u	u	l	l	l	u
Knight (1990)	51	h	h	h	u	u	l	l	l	u
Fogarty et al (1984)	52	h	h	h	u	u	u	l	h	h
Atkins (1986)	73	l	l	l	u	u	l	l	l	l
Cloete and Heydenrych (1986)	74	l	l	l	u	u	l	l	l	l
Fahmy and Dufour (1988)	75	l	l	u	u	u	l	l	h	u
Long et al (1989)	84	l	l	u	u	u	l	l	h	u
Brash et al (1994b)	118	l	l	l	u	u	u	u	l	u
Brash et al (1994c)	119	l	l	l	u	u	u	u	l	u
Fogarty et al (1994)	120	l	l	l	u	u	l	l	l	l
Meyer et al (1994)	125	l	l	l	u	u	l	l	l	l
van Haandel & Visscher (1995)	144	l	l	l	u	u	l	l	l	l
Thieme et al (1999)	167	h	h	h	u	u	u	u	u	h
da Silva et al (2000)	174	h	h	h	h	h	h	h	h	h
Swan et al (2001)	189	l	l	l	u	u	l	l	l	l
Hanford et al (2002)	196	l	l	l	u	u	l	l	l	l
Ozcan et al (2002)	199	h	h	h	u	u	u	u	u	l
Southey et al (2002)	202	l	l	l	u	u	l	l	l	u
Hanford et al (2003)	204	l	l	l	u	u	l	l	l	l
Ingham (2005)	205	l	l	l	u	u	l	l	l	l

SYRCLE Risk of Bias Assessment: l=low risk of bias, h=high risk of bias, u=unclear-insufficient details. Key for Headings: RS = Random sequence; AC = Allocation concealment; BS = Baseline similarity; BC = Blinding of caregivers; BO = Blinding of outcomes; IO = Incomplete outcomes; SR = Selective reporting; OB = Other bias (nutrition, climate, management); OJ = Overall judgement.

Table S6a. Sensitivity analyses: omnibus test statistics and variance (R^2) for reproductive components.

Outcome	Model	*F* (df)	*P-value*	R^2
Fertility	Unweighted	1.44 (4,107)	0.227	0.051
	Weighted	4.88 (4,107)	0.001	0.154
Fecundity	Unweighted	55.80 (4,107)	<0.001	0.676
	Weighted	37.55 (4,107)	<0.001	0.584
Lamb survival	Unweighted	4.86 (4,107)	0.001	0.154
	Weighted	4.96 (4,107)	0.001	0.157
Lambs weaned	Unweighted	10.27 (4,107)	<0.001	0.278
	Weighted	14.26 (4,107)	<0.001	0.348

Table S6b. Sensitivity analyses: estimated marginal means (± SE) for each breed class under both weighting schemes.

		Unweighted				Weighted			
**Outcome**	**Breed class**	**Mean**	**± SE**	**CI_-low**	**CI_-high**	**Mean**	**± SE**	**CI_-low**	**CI_-high**
Fertility	1	80.70	1.71	77.32	84.09	81.25	0.96	79.35	83.16
Fertility	2	85.92	1.58	82.79	89.04	87.49	1.06	85.39	89.60
Fertility	3	83.74	2.03	79.70	87.77	83.21	1.59	80.05	86.36
Fertility	4	87.20	5.75	75.80	98.60	87.47	16.99	53.78	121.15
Fertility	5	85.47	3.00	79.52	91.43	82.03	4.34	73.42	90.64
Fecundity	1	128.29	3.66	121.03	135.54	126.37	2.14	122.14	130.61
Fecundity	2	140.21	3.37	133.52	146.89	139.48	2.36	134.80	144.17
Fecundity	3	136.33	4.36	127.70	144.97	120.99	3.55	113.95	128.02
Fecundity	4	124.00	12.32	99.58	148.42	114.53	37.86	39.48	189.58
Fecundity	5	234.50	6.43	221.75	247.25	239.64	9.68	220.45	258.82
Lamb survival	1	80.75	1.58	77.63	83.88	79.67	0.89	77.90	81.44
Lamb survival	2	81.91	1.45	79.03	84.79	78.35	0.99	76.39	80.31
Lamb survival	3	83.16	1.88	79.44	86.88	86.03	1.49	83.08	88.97
Lamb survival	4	87.03	5.30	76.52	97.55	80.14	15.84	48.75	111.54
Lamb survival	5	69.76	2.77	64.26	75.25	76.96	4.05	68.94	84.99
Lambs weaned	1	83.95	4.21	75.59	92.30	89.76	0.03	89.70	89.81
Lambs weaned	2	98.77	3.89	91.07	106.47	114.88	0.02	114.84	114.91
Lambs weaned	3	97.06	5.02	87.12	107.01	87.19	0.05	87.09	87.30
Lambs weaned	4	95.10	14.19	66.97	123.23	80.43	0.59	79.27	81.58
Lambs weaned	5	138.50	7.41	123.81	153.19	150.34	0.15	150.04	150.63

Breed classes: 1= wool, 2 = dual purpose, 3 = meat, 4 = milk, and prolific = 5. Note: Breed class 4 had a small sample size (n = 3), resulting in wider standard errors; contrasts involving this group should be interpreted cautiously.

Table S7. Weighted means ± SEM (*n*) for fertility (ewes pregnant of ewes exposed to rams), fecundity (lambs born of ewes pregnant), lamb survival (lambs present at weaning of lambs born) and lambs weaned (lambs weaned of ewes exposed to rams) categorised according to breed when all reproductive components are present.

Breed	*n*	Fertility	Fecundity	Lamb survival	Lambs weaned
ABRO Finn synthetic	1	86.0 ± 11.64	169.0 ± 24.33	81.1 ± 7.30	117.9 ± 24.39
Afar	1	90.0 ± 23.79	106.0 ± 49.74	75.0 ± 14.93	72.0 ± 49.86
Akkaraman	1	91.7 ± 8.49	102.4 ± 17.75	93.5 ± 5.33	87.8 ± 17.79
Akkaraman x Merino	1	92.2 ± 43.09	127.1 ± 90.09	85.3 ± 27.04	100 ± 90.31
Awassi	1	89.8 ± 40.21	120.0 ± 84.07	84.5 ± 25.23	91.1 ± 84.27
Barki	1	79.0 ± 5.23	104.7 ± 10.94	79.2 ± 3.28	65.5 ± 10.96
Bonga	1	95.0 ± 19.12	145.0 ± 39.98	95.0 ± 12.00	131.0 ± 40.08
Border Leicester x Merino	1	92.1 ± 20.26	166.0 ± 42.36	79.6 ± 12.71	121.7 ± 42.46
Chios x Kivircik	1	88.0 ± 97.50	141.0 ± 203.86	100.0 ± 61.18	124.1 ± 204.35
Columbia	2	86.0 ± 1.95	138.5 ± 4.07	71.2 ± 1.22	84.8 ± 4.07
Corriedale	4	91.2 ± 5.44	136.3 ± 11.37	84.2 ± 3.41	106.1 ± 11.40
Crioula	1	75.0 ± 12.70	120.0 ± 26.55	70.0 ± 7.97	63.0 ± 26.61
D’Man	1	94.4 ± 9.11	238.0 ± 19.05	85.3 ± 5.72	191.6 ± 19.10
DLS Composite	1	86.3 ± 39.80	144.0 ± 83.23	83.5 ± 24.98	103.8 ± 83.42
Dorper	3	80.0 ± 2.35	123.1 ± 4.91	88.0 ± 1.48	86.6 ± 4.93
Dorset	2	90.7 ± 4.73	132.3 ± 9.89	84.1 ± 2.97	101.1 ± 9.92
East Friesian x Corriedale	1	83.6 ± 29.18	125.0 ± 61.02	86.1 ± 18.31	89.2 ± 61.17
Finn	3	71.2 ± 13.74	252.5 ± 28.74	55.2 ± 8.62	99.2 ± 28.81
Flevolander	1	72.8 ± 7.68	268.0 ± 16.05	81.0 ± 4.82	158.0 ± 16.09
Hyfer	1	79.3 ± 12.12	146.0 ± 25.33	85.7 ± 7.60	99.2 ± 25.39
Kivircik	1	75.0 ± 92.12	133.0 ± 192.63	92.9 ± 57.81	92.6 ±193.09
Lori-Bakhtiari	1	90.0 ± 6.65	117.0 ± 13.91	89.3 ± 4.17	94.0 ± 13.94
Makooei	1	93.0 ± 8.34	116.0 ± 17.44	84.5 ± 5.23	91.1 ± 17.48
Maternal Australia	1	68.0 ± 19.52	176.0 ± 40.80	81.0 ± 12.25	99.0 ± 40.90
Merino FecB+	1	83.2 ± 24.65	235.5 ± 51.55	39.4 ±15.47	76.9 ± 51.67
Merino fine	5	79.8 ± 2.18	115.1 ± 4.57	81.5 ± 1.37	74.4 ± 4.58
Merino medium	13	83.0 ± 1.77	138.5 ± 3.70	79.6 ± 1.11	90.6 ± 3.71
Merino strong	4	81.2 ± 5.48	135.1 ± 11.46	79.3 ± 3.44	88.3 ± 11.49
Merino various	13	80.8 ±1.53	124.6 ± 3.21	79.2 ± 0.96	79.7 ± 3.21
Perendale	1	91.0 ± 18.08	147.0 ± 37.80	84.4 ± 11.35	112.9 ± 37.89
Perendale Fec++	1	94.0 ± 30.65	143.0 ± 64.08	87.4 ± 19.23	117.5 ± 64.24
Perendale FecB+	1	94.0 ± 27.87	260.0 ± 58.27	62.3 ± 17.49	152.3 ± 58.41
Polwarth	2	87.2 ± 12.37	114.7 ± 25.87	76.2 ± 7.77	75.7 ± 25.94
Rambouillet	4	77.3 ± 4.36	127.7 ± 9.12	84.4 ± 2.74	83.2 ± 9.14
Rambouillet Fec++	1	96.0 ± 80.14	161.0 ± 167.57	95.1 ± 50.29	147.0 ± 167.97
Rambouillet FecB+	1	92.6 ± 50.01	266.0 ± 104.58	75.4 ± 31.38	185.7 ±104.83
Romanov	1	88.2 ± 35.74	176.0 ± 74.74	88.6 ± 22.43	137.6 ± 74.92
Romney	5	93.6 ± 2.48	136.0 ± 5.19	88.7 ± 1.56	113.4 ± 5.20
Romney Fec++	1	93.0 ± 37.29	140.0 ± 77.95	87.1 ± 23.39	113.4 ± 78.13
Romney FecB+	1	93.0 ± 34.91	254.0 ± 72.99	62.2 ± 21.91	146.9 ± 73.17
Romney x Border Leicester	4	88.2 ± 7.24	127.9 ± 15.13	82.8 ± 4.54	93.6 ± 15.17
Sabi	1	88.0 ± 7.75	116.0 ± 16.20	87.0 ± 4.86	88.8 ± 16.24
Santa Ines x Crioula	1	42.0 ± 22.25	119.0 ± 46.52	38.0 ±13.96	19.0 ± 46.64
Scottish Blackface	1	85.8 ± 6.51	109.4 ± 13.61	83.7 ± 4.08	78.8 ± 13.64
Suffolk	3	77.4 ± 22.75	160.3 ± 47.58	75.0 ± 14.28	93.6 ± 47.69
Suffolk x Targhee	2	89.9 ± 27.47	151.7 ± 57.43	84.5 ± 17.24	115.0 ± 57.57

Targhee	4	87.8 ± 2.56	150.1 ± 5.14	73.6 ± 1.54	97.3 ± 5.15
Targhee x Suffolk	2	84.6 ± 34.56	171.8 ± 72.26	83.6 ± 21.68	121.1 ± 72.43
Tazegzawt	1	87.0 ± 59.55	182.9 ± 124.53	88.0 ± 37.37	140.0 ± 124.82
Tsogai	1	82.8 ± 26.06	145.0 ± 54.48	79.6 ± 16.35	95.6 ± 54.61
Turcan	2	95.9 ± 39.67	119.2 ± 82.95	92.3 ± 24.89	105.7 ± 83.15
Turcan x Blueface Leicester	2	95.7 ± 38.18	171.9 ± 79.84	94.2 ± 23.96	155.3 ± 80.03

Supplementary Figures

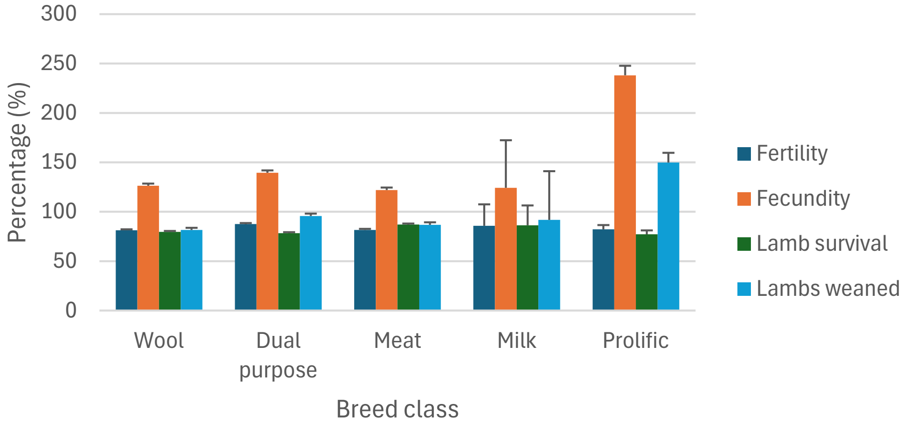

Figure S1. Weighted means ± SEM (*n*) for fertility (ewes pregnant of ewes exposed to rams), fecundity (lambs born of ewes pregnant), lamb survival (lambs present at weaning of lambs born) and lambs weaned (lambs weaned of ewes exposed to rams) categorised according to breed class.

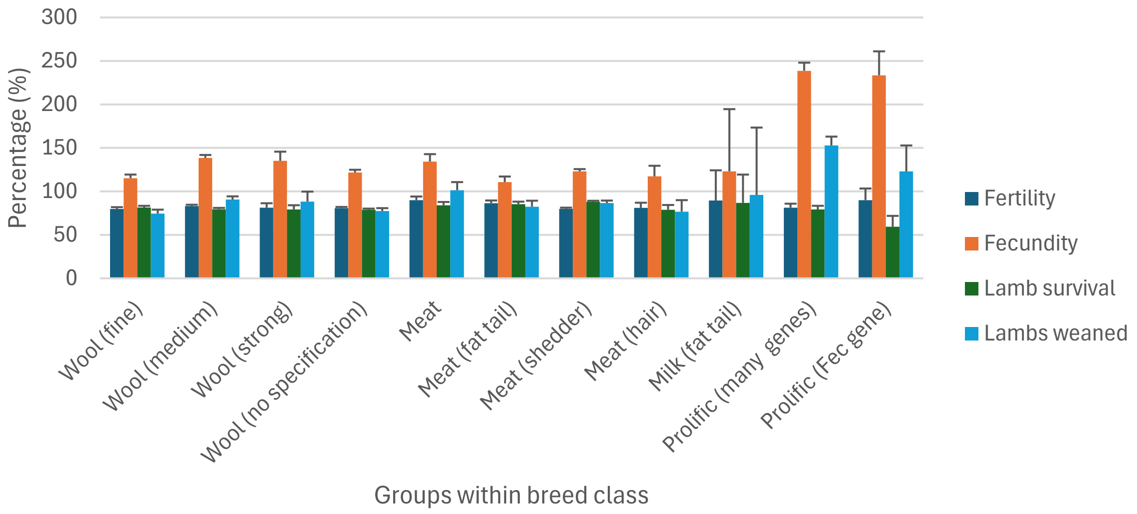

Figure S2. Weighted means ± SEM for fertility (ewes pregnant of ewes exposed to rams), fecundity (lambs born of ewes pregnant), lamb survival (lambs present at weaning of lambs born) and lambs weaned (lambs weaned of ewes exposed to rams) of breed class and group (wool – fine, medium, strong, unknown; meat – narrow tail, fat tail, shedder, hair; milk – narrow tail, fat tail; prolific – many genes, Fec gene).

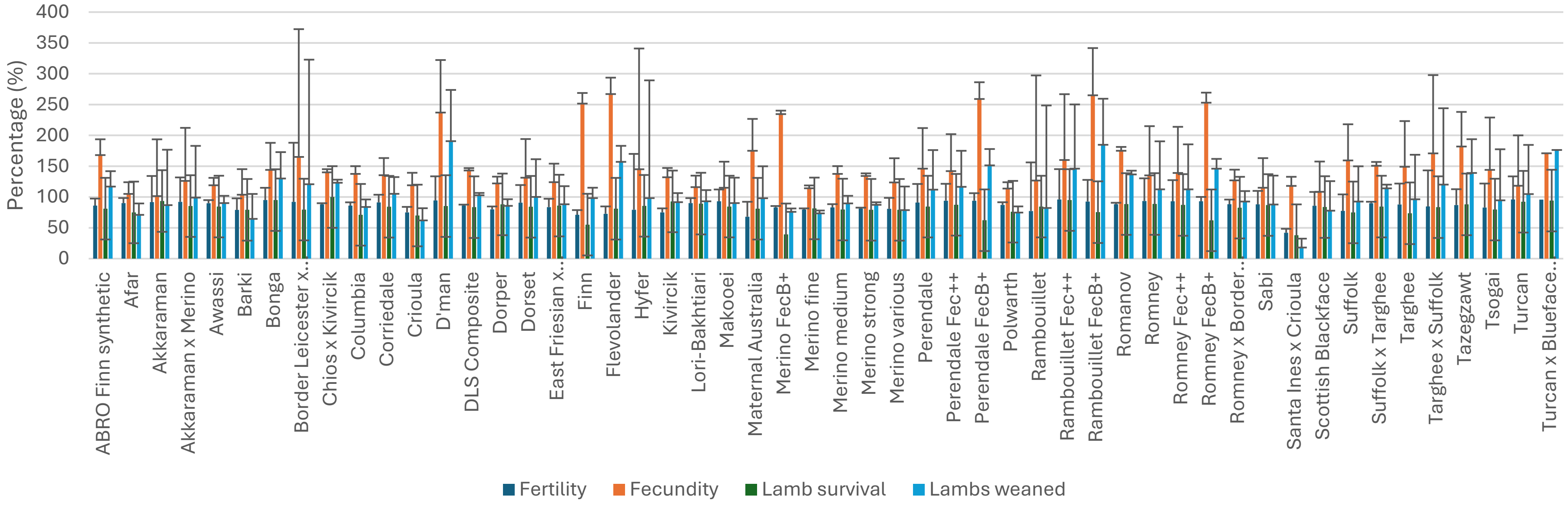

Figure S3. Weighted means ± SEM for fertility (ewes pregnant of ewes exposed to rams), fecundity (lambs born of ewes pregnant), lamb survival (lambs present at weaning of lambs born) and lambs weaned (lambs weaned of ewes exposed to rams) of breeds.

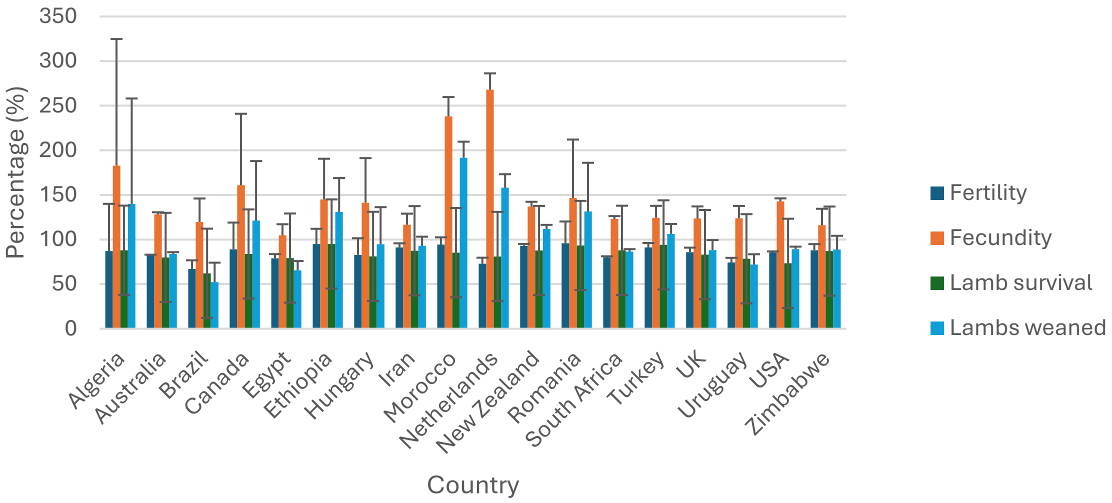

Figure S4. Weighted means ± SEM for fertility (ewes pregnant of ewes exposed to rams), fecundity (fetuses/lambs born of ewes pregnant), lamb survival (lambs present at weaning of lambs born) and lambs weaned (lambs weaned of ewes exposed to rams) of breeds categorised by country.

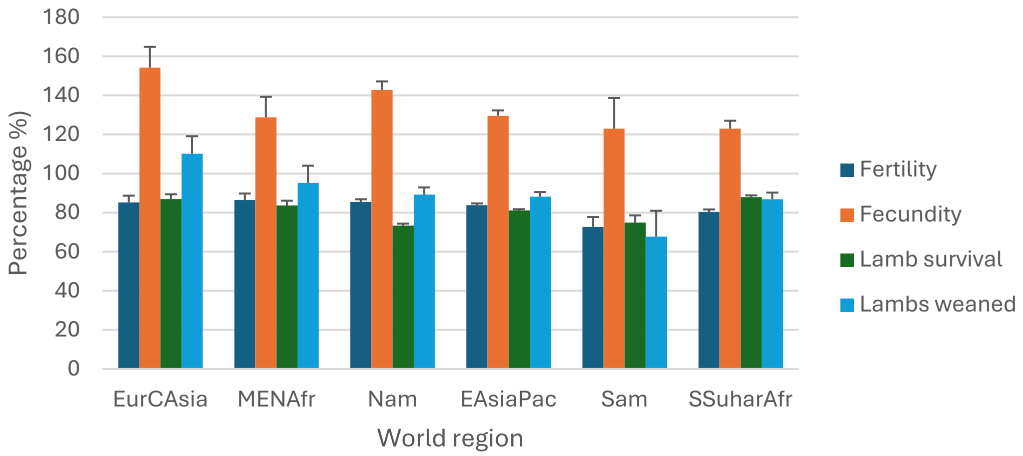

Figure S5. Weighted means ± SEM for fertility (ewes pregnant of ewes exposed to rams), fecundity (lambs born of ewes pregnant), lamb survival (lambs present at weaning of lambs born) and lambs weaned (lambs weaned of ewes exposed to rams) of breeds categorised by world region. World regions: Europe (EurCAsia; including Central Asia); Middle East and North Africa (MeNAfr); North America (NAm; including central America and Greenland); East Asia and Pacific (EAPac); South America (SAm); Sub-Saharan Africa (SSuharAfr).
